# Mitochondrial Plasticity Promotes Resistance to Sorafenib and Vulnerability to STAT3 Inhibition in Human Hepatocellular Carcinoma

**DOI:** 10.3390/cancers13236029

**Published:** 2021-11-30

**Authors:** Shusil K. Pandit, Giada Sandrini, Jessica Merulla, Valentina Nobili, Xin Wang, Alessia Zangari, Andrea Rinaldi, Dheeraj Shinde, Giuseppina M. Carbone, Carlo V. Catapano

**Affiliations:** Tumor Biology and Experimental Therapeutics Program, Institute of Oncology Research, Università della Svizzera italiana, 6500 Bellinzona, Switzerland; shusilpandit@gmail.com (S.K.P.); giada.sandrini@ior.usi.ch (G.S.); jessica.merulla@ior.iosi.ch (J.M.); nobiliv@hotmail.com (V.N.); xiaowuxian2006@126.com (X.W.); alessia.zangari@ior.usi.ch (A.Z.); andrea.rinaldi@ior.usi.ch (A.R.); Dheeraj.shinde@ior.usi.ch (D.S.); pina.carbone@ior.usi.ch (G.M.C.)

**Keywords:** liver cancer, hepatocellular carcinoma, sorafenib, tyrosine kinase inhibitors, drug resistance, mitochondria, mitochondrial protein translation, mitochondrial ribosomal proteins, STAT3, OPB-111077

## Abstract

**Simple Summary:**

Enhanced expression of mitochondrial ribosomal proteins and marked reprogramming of the mitochondrial network are associated with sorafenib resistance in human cell lines and hepatocarcinoma patients, providing novel actionable targets for increasing therapeutic efficacy.

**Abstract:**

The multi-kinase inhibitor sorafenib is a primary treatment modality for advanced-stage hepatocellular carcinoma (HCC). However, the therapeutic benefits are short-lived due to innate and acquired resistance. Here, we examined how HCC cells respond to sorafenib and adapt to continuous and prolonged exposure to the drug. Sorafenib-adapted HCC cells show a profound reprogramming of mitochondria function and marked activation of genes required for mitochondrial protein translation and biogenesis. Mitochondrial ribosomal proteins and components of translation and import machinery are increased in sorafenib-resistant cells and sorafenib-refractory HCC patients show similar alterations. Sorafenib-adapted cells also exhibited increased serine 727 phosphorylated (pSer727) STAT3, the prevalent form in mitochondria, suggesting that STAT3 might be an actionable target to counteract resistance. Consistently, a small-molecule STAT3 inhibitor reduces pSer727, reverts mitochondrial alterations, and enhances the response to sorafenib in resistant cells. These results sustain the importance of mitochondria plasticity in response to sorafenib and identify a clinically actionable strategy for improving the treatment efficacy in HCC patients.

## 1. Introduction

Hepatocellular carcinoma (HCC) is the sixth most common cancer and the second leading cause of cancer deaths worldwide [1]. HCC pathogenesis involves sustained inflammation and multiple genetic and epigenetic alterations that account for the high degree of molecular and phenotypic heterogeneity [2]. Advanced-stage HCC patients with extensive liver involvement and extrahepatic dissemination receive systemic therapies [2]. In the last decade, the primary drug for advanced-stage HCC has been the tyrosine kinase inhibitor (TKI) sorafenib [3]. However, the clinical benefits are modest, with a median overall survival (OS) increase of only three months [4]. Other TKIs such as regorafenib, lenvatinib, and cabozantinib have also shown efficacy [3]. Immune checkpoint inhibitors have promising activity in HCC, but no clear advantage in OS over standard treatment has been seen thus far in randomized studies [5]. Lately, combined immune checkpoint inhibition and VEGF blockade with atezolizumab and bevacizumab have significantly improved OS in advanced HCC [6]. However, despite these recent successes, many promising drugs have failed to demonstrate durable therapeutic responses and significant survival advantages in the clinic [3]. Thus, new approaches are required including novel combinatorial strategies and biomarkers to select potentially responsive patients. In this context, a greater understanding of the biological mechanisms underlying the short duration of responses and the almost inevitable emergence of resistance to TKI treatment in HCC patients could be highly relevant.

Sorafenib inhibits multiple kinases including RAF, vascular endothelial growth factor receptor (VEGFR), platelet-derived growth factor receptor (PDGFR), and KIT [3,7]. It blocks tumor cell proliferation, survival, and angiogenesis, thus interfering with critical processes in hepatocarcinogenesis [3,7]. Despite the tremendous therapeutic potential, most HCCs are either innately refractory or acquire resistance to sorafenib, leading to rapid disease progression [8]. At the cellular level, the emergence of resistance may imply the activation of compensatory signaling pathways and phenotypic transitions with the acquisition of mesenchymal and stem cell-like features that enhance the survival and resilience of cancer cells [9,10,11]. However, the clinical relevance of these mechanisms is unclear, and strategies to identify and overcome innate and acquired resistance remain elusive [8].

The emergence of a resistant phenotype involves complex rewiring of epigenetic, transcriptional, and cellular signaling pathways that, in an adaptive process, reprogram tumor cells to cope with treatment-induced stress [12,13,14]. Metabolic changes are also essential for tumorigenesis, as cancer cells can rapidly adapt to face metabolic stress [15]. Reprogramming of cell metabolism and increased reliance on mitochondrial activity are vital features in the transition states, leading to mesenchymal and cancer stem cell properties [16,17,18]. Indeed, enhanced mitochondrial oxidative phosphorylation (OXPHOS) marks tumor subtypes with reduced response to treatment [19,20,21], and targeting mitochondrial processes is a promising strategy to prevent treatment resistance with several compounds already in clinical trials [22,23].

Signal transducer and activator of transcription 3 (STAT3) is a crucial transcription factor (TF) in multiple oncogenic pathways and has an essential role in HCC pathogenesis [24,25]. Interestingly, activation of STAT3 has emerged as a mechanism of resistance to multiple TKIs in various cancer models and inhibiting STAT3 increases treatment efficacy [26]. Signaling through the IL-6/JAK pathway induces phosphorylation of STAT3 at tyrosine 705 (Tyr705), leading to nuclear translocation and transcriptional activation of STAT3 target genes [25]. Conversely, phosphorylation at serine 727 (Ser727) by protein serine kinases enhances mitochondrial translocation, which is essential for carcinogenesis due to its role in promoting a metabolic switch and protecting against metabolic stress [25,27,28]. However, presently, there is limited evidence connecting mitochondrial STAT3 to resistance to anticancer drugs.

In this study, we sought to define the mechanisms that contribute to sorafenib resistance in HCC. We exposed human HCC cells to sorafenib in bulk cell cultures and clonal growth conditions to derive a clinically relevant model. Surviving HCC cells adapted to the prolonged drug exposure and reduced their sensitivity to sorafenib. Here, we describe the underpinnings of this adaptive response, which involves broad transcriptional changes, profound reprogramming of the mitochondrial network, and non-canonical activation of STAT3 by Ser727 phosphorylation. Our data reveal the impact of mitochondrial plasticity in sorafenib resistance and point to clinically relevant options for detecting potentially responsive patients and improving the treatment of HCC.

## 2. Materials and Methods

### 2.1. Cell Lines

Huh7, HepG2, SNU-387, and SNU-423 cell lines were purchased from ATCC and JCRB and maintained in DMEM (Gibco) supplemented with 10% FBS. Sorafenib-resistant Huh7 cells were generated by treatment with increasing concentrations of sorafenib and kept in the presence of sorafenib (5 µM) for up to 6–9 months. Huh7 cells with inducible knockdown of STAT3 (I-shSTAT3 Sorafenib-R) were generated by transduction with IPTG-inducible lentiviral shRNA (I-shSTAT3) (Sigma, Darmstadt, Germany) followed by the selection of transduced cells in the presence of 5 µg/mL puromycin for ten days. GRIM19 (NDUFA13) was knocked down by transducing cells with lentiviral shRNA (Sigma), followed by selection with puromycin for ten days. OPB-111077 was a gift from Otsuka Pharmaceuticals. Sources of all other reagents are indicated in the Supplementary Materials.

### 2.2. Gene Expression Profiling

Total RNA was isolated using the RNeasy Kit (Qiagen). RNA samples were processed using the Illumina HumanHT-12 v4 BeadChip Kit, and microarrays were scanned on an Illumina HiScan SQ system. Data were extracted using Illumina GenomeStudio software, imported in Genomics Suite 6.4 (Partek Incorporated, Saint Louis, MO, USA), and quantile normalized. Differential expression analysis of transcripts was conducted by analysis of variance (ANOVA). Functional annotation analysis of upregulated and downregulated transcripts was performed with Enrichr using the available tools under the Transcription, Pathways, Ontologies, and Disease/Drugs paths [29]. Specific gene sets were downloaded from MSigDB (http://www.broadinstitute.com, accession date: 19 June 2020).

Detailed materials and methods are available in the Supplementary Materials.

## 3. Results

### 3.1. Adaptation of HCC Cells to Chronic Exposure to Sorafenib

**Figure 1:**
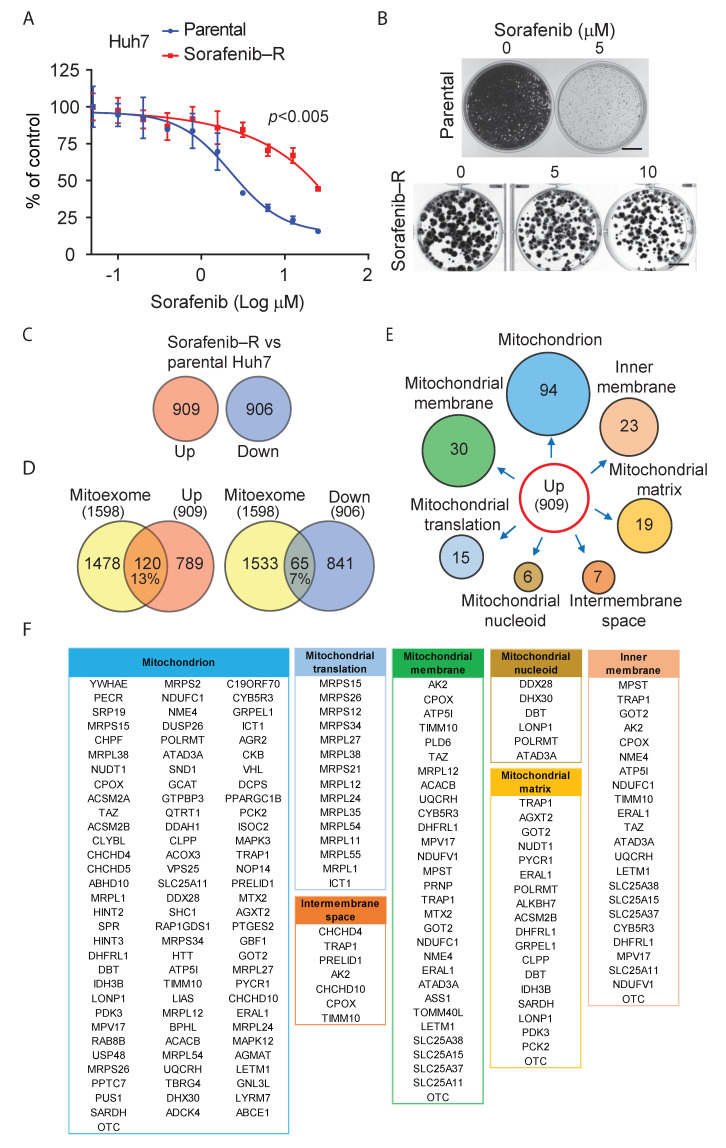
Extensive transcriptional and mitochondrial reprogramming in sorafenib-resistant hepatocarcinoma cells. (**A**) Proliferation of parental and sorafenib-resistant (sorafenib-R) Huh7 cells in the presence of sorafenib. Cell viability was measured after 72 h with the MTT assay. Sorafenib-R cells were generated by 3-month continuous growth with sorafenib. (**B**) Growth of parental (top) and sorafenib-resistant (bottom) Huh7 cells exposed to sorafenib. Sorafenib-R cells were generated after 3-weeks of continuous exposure to sorafenib in clonal growth conditions. Bar, 10 mm. (**C**) Genes up- and downregulated in sorafenib-resistant Huh7 cells compared to parental cells. (**D**) Overlap of genes up- and downregulated in sorafenib-resistant Huh7 cells with the human mitoexome. (**E**) Numbers and functional classes of mitochondrial genes upregulated in sorafenib-resistant Huh7 cells. (**F**) Mitochondrial genes upregulated genes in sorafenib-resistant Huh7 cells.

We examined the effects of sorafenib in a panel of human HCC cell lines. Sorafenib reduced the growth of SNU-387, SNU-423, HepG2, and Huh7 cells in cell proliferation and clonogenic assays, with Huh7 cells showing slightly higher sensitivity (Supplementary Figure S1A,B). To investigate the mechanism of adaptive resistance to the drug, we continuously exposed bulk cultures of HCC cells to cytotoxic concentrations (5–10 μM) of sorafenib. After an initial phase of growth arrest, HCC cells adapted and started to grow again within 3–5 weeks despite the continuous addition of the drug. After 3–6 months, continuously treated Huh7 cells exhibited significantly reduced sensitivity to sorafenib with IC_50_ values increasing from 2.8 to 11.7 μM (*p*-value ≤ 0.005) compared to parental Huh7 cells (Figure 1A). We observed this phenomenon in multiple independent experiments using the same protocol, generating various sublines that grew undisturbed in the presence of sorafenib.

Prolonged exposure of Huh7 cells to sorafenib in clonal growth conditions led similarly to the rapid emergence of sorafenib-resistant clones (Figure 1B and Supplementary Figure S2A). After three weeks, cells derived from the surviving clones were highly resistant to concentrations of sorafenib that suppressed the growth of parental Huh7 cells. The phenomenon was highly consistent, suggesting that this model could provide valid information on the adaptation mechanisms after prolonged exposure to the drug. Sorafenib-resistant cells grew slightly slower than parental Huh7 cells in adherent cell cultures (Supplementary Figure S2B). However, resistant and parental Huh7 cells had similar tumor-sphere forming ability in 3D cultures, indicating that they retained stemness and tumor-initiating capability (Supplementary Figure S2C). Moreover, there was no difference in the capacity of parental and resistant cells to grow as subcutaneous tumor xenografts in nude mice (Supplementary Figure S2D).

### 3.2. Deregulation of Mitochondrial Protein Translation and Import in Sorafenib-Adapted Huh7 Cells

To obtain insights into the biological processes underlying the acquisition of sorafenib resistance, we examined the transcriptome of parental and sorafenib-resistant Huh7 cells. There were about 900 genes (*p*-value ≤ 0.05) upregulated and downregulated in resistant cells (Figure 1C; Supplementary dataset S1 and S2). Pathway enrichment analysis revealed a striking prevalence (adjusted *p*-value ≤ 0.005) of mitochondrial translation and RNA processing among upregulated genes (Table 1). Gene Ontology enrichment analysis consistently indicated mitochondria, ribosomes, and nucleolus as the most represented components among upregulated genes (adjusted *p*-value ≤ 0.05; Supplementary Table S1). Conversely, platelets and endoplasmic reticulum prevailed among downregulated genes (adjusted *p*-value ≤ 0.05; Table 1 and Supplementary Table S1). Notably, a significant fraction of activated (*n* = 120, 13.2%) and repressed (*n* = 65, 7.2%) genes were part of the mitoexome [30] (i.e., nuclear-encoded mitochondrial proteins) (Figure 1D). Furthermore, Myc and Myc-cofactor Max were among the top enriched TFs associated with genes activated in sorafenib-resistant Huh7 cells (adjusted *p*-value ≤ 0.005; Table 2; Supplementary Table S2). Indeed, Myc has a central role in controlling nuclear-encoded mitochondrial proteins, with Myc targets representing up to 35% of the mitoexome [31]. Conversely, downregulated genes showed a prevalence of putative targets of SOX2, a pluripotency TF (adjusted *p*-value ≤ 0.005; Table 2; Supplementary Table S3).

We performed enrichment analysis of drug perturbation datasets to search for drug-induced transcriptional changes matching the transcriptional profile of sorafenib-resistant Huh7 cells. We found a significant overlap between genes upregulated in sorafenib-resistant cells and those repressed by the RAF kinase inhibitor vemurafenib (adjusted *p*-value ≤ 0.005; Supplementary Table S4). There was also significant overlap between genes induced by the EGFR inhibitor cetuximab and those upregulated in sorafenib-resistant cells. In contrast, the genes downregulated in sorafenib-resistant cells exhibited an inverse relation. Interestingly, both RAF/MAPK and EGFR signaling are involved in sorafenib resistance [8]. Thus, along with the activation of sorafenib-related signal transduction pathways, the transcriptomic analysis revealed the deregulation of mitochondrial processes as a significant contributor to the resistant phenotype in Huh7 cells. 

Examining the biological functions of the genes upregulated in sorafenib-resistant cells, we found a striking enrichment of genes associated with mitochondrial protein translation (MPT), mitochondrial membranes, and protein import components (Figure 1E,F). Notably, sorafenib-resistant cells had upregulated several constituents of the large (MRPL) and small (MRPS) mitochondrial ribosomal subunits [32]. Members of the CHCH domain (CHCHCD)-containing protein family such as CHCHD10, CHCHD4, and CHCHD5, essential for importing nuclear-encoded proteins in mitochondria [33], were also overexpressed. Thus, the main features characterizing sorafenib-resistant cells were enhanced protein translation and import in mitochondria, vital elements for mitochondria biogenesis and constitution of a functional mitochondrial network.

### 3.3. Mitochondrial Ribosomal Protein Expression and Response to Sorafenib in HCC Patients

**Figure 2:**
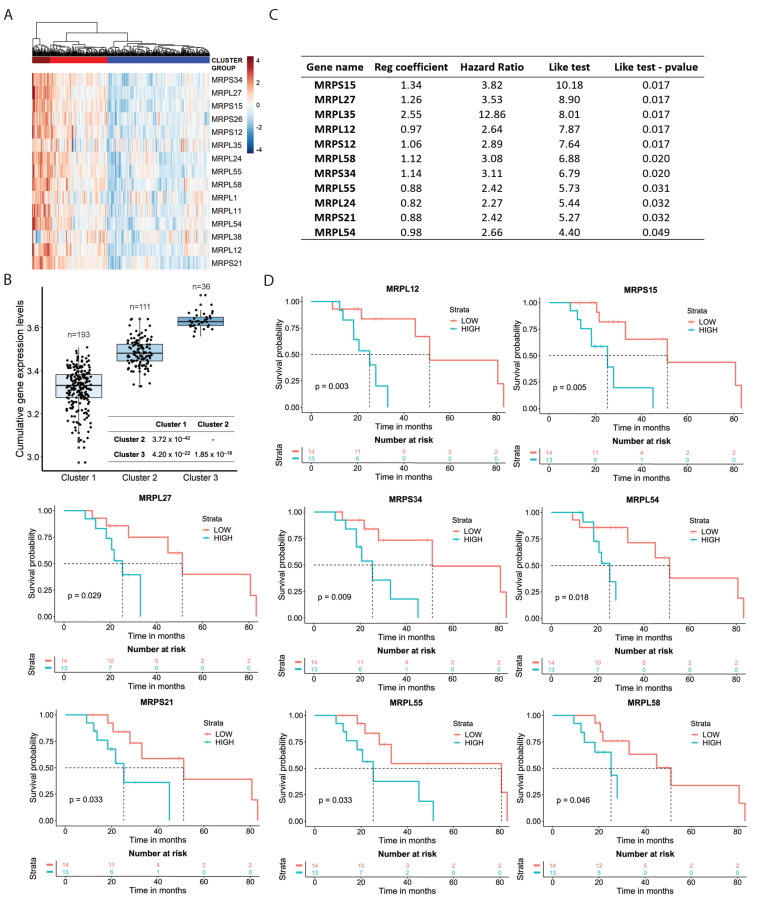
Expression of mitochondrial ribosomal proteins and response to sorafenib in hepatocarcinoma patients. (**A**) Un–supervised clustering of HCC samples in the TCGA patient cohort based on the expression of the sorafenib upregulated mitochondrial ribosomal protein (MRP) gene set. Total number of patients examined was 340. Number of patients in each cluster is reported in B. (**B**) Cumulative expression of MRP gene set in the distinct clusters of HCC samples. Bottom, *p*-values for inter−cluster comparisons. (**C**) Association of individual MRP gene expression and overall survival by Cox proportional hazard regression analysis. (**D**) Kaplan–Meyer survival analysis of overall survival based on the median expression of individual MRP genes in subset of patients receiving sorafenib in the TCGA cohort.

Transcriptomic analysis of sorafenib-resistant cells provided novel clues into the adaptation mechanism to prolonged exposure to the drug, pointing to increased expression of multiple nuclear-encoded mitochondrial components essential for mitochondrial protein translation and import. To verify the clinical relevance of this finding, we asked whether human HCC samples had an abnormal expression of mitochondria-related genes and whether there was a relationship with the clinical response to sorafenib. To this end, we interrogated a large TCGA dataset (https://www.cancer.gov/about-nci/organization/ccg/research/structural-genomics/tcga, accession date: 21 July 2020) [34] that included 50 normal liver and 371 HCC samples, with 340 cases having a complete stage and survival information (Supplementary Figure S3A). Only a limited subset of the patients (*n* = 27) had documented evidence of treatment with sorafenib. Interestingly, the expression of the MRP genes upregulated in sorafenib-resistant cells divided tumor samples into distinct clusters with significant differences in cumulative MRP gene expression (Figure 2A,B; Supplementary Figure S3B). We verified the reproducibility of this finding in an independent cohort of HCC patients (ICGC, https://dcc.icgc.org/projects/LIRI-JP, accession date: 21 July 2020) that included 195 normal liver and 240 HCC samples. We obtained a similar clustering of HCC samples based on the cumulative expression of the MRP genes (Supplementary Figure S3C). Notably, the level of MRP gene expression was associated with reduced overall survival in the subgroup of sorafenib-treated patients in the TCGA cohort (*p*-value: 0.024; Supplementary Figure S3D). In contrast, MRP expression did not affect the survival of the total patient population (Supplementary Figure S3E). In a multivariate analysis, the cumulative expression of the MRP gene set was predictive of overall survival in the sorafenib-treated group (*p*-value: 0.008), whereas the stage was not a predictive covariate (*p*-value = 0.065) (Supplementary Figure S3F).

Considering all MRP genes, we found that expression of many individual MRPs was significantly associated with overall survival (*p*-value ≤ 0.05) in sorafenib-treated patients (Supplementary Figure S4A). However, none had significant predictive value in the total HCC patient population (Supplementary Figure S4B). Next, using a feature selection approach based on the Cox proportional hazard method, we found that 11 MRP genes among the 15 upregulated in sorafenib-resistant cells were predictive of overall survival in sorafenib-treated patients (Figure 2C). Kaplan–Meyer survival analysis based on the median gene expression showed that *MRPL12*, *MRPS15*, *MRPS34*, *MRPL54*, *MRPS21*, *MRPL27*, *MRPL58,* and *MRPL55* were reliable predictors of overall survival, with higher expression associated with worse clinical outcomes (Figure 2D). Thus, these data revealed increased expression of mitochondrial ribosomal proteins is frequent in human HCCs and makes tumors less likely to respond to sorafenib.

### 3.4. Enhanced Mitochondrial Biogenesis in HCC Cells Resistant to Sorafenib

**Figure 3:**
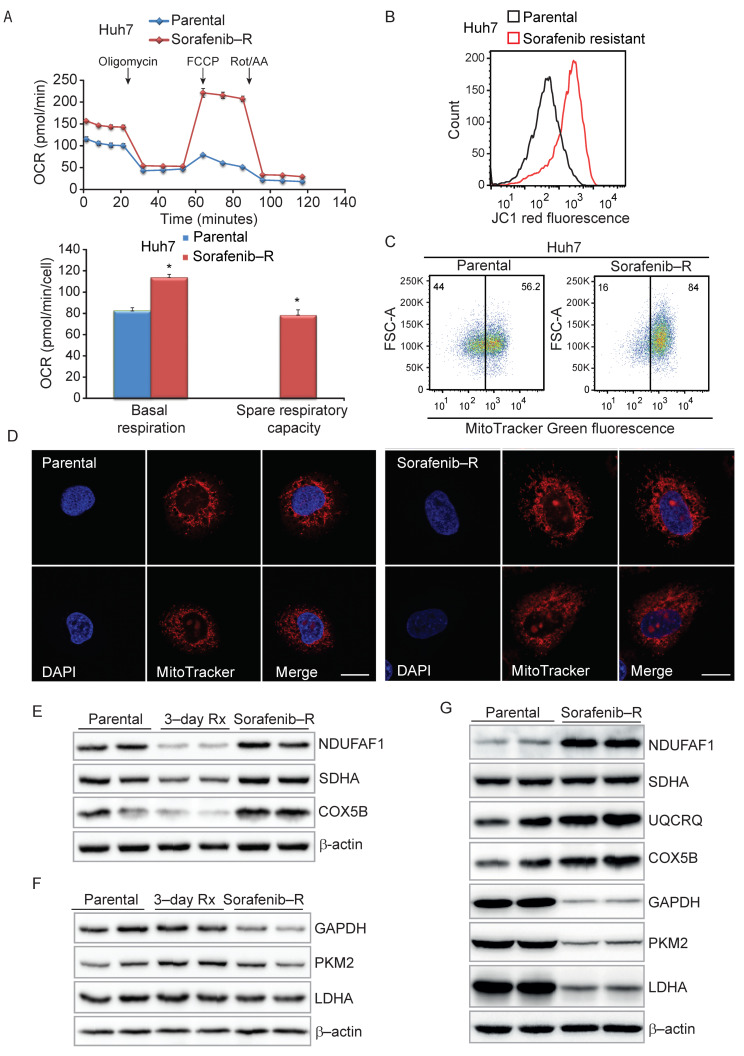
Increased mitochondrial activity in sorafenib resistant Huh7 cells. (**A**) Mitochondrial oxygen consumption rate (OCR) in sorafenib-resistant and parental Huh7 cells determined by Seahorse XFp analyzer and mitochondrial stress test. (**Top**) Real-time OCR measurements normalized to cell number. (**Bottom**) Basal and spare respiratory capacity based on OCR profiles. *, *p* ≤ 0.005. (**B**) Mitochondrial membrane potential (MMP) assessed by JC-1 staining (JC-1 red fluorescence) and flow cytometry in sorafenib-resistant and parental Huh7 cells. (**C**) MitoTracker Green staining and mitochondrial mass assessment by flow cytometry in sorafenib-resistant and parental Huh7 cells. (**D**) Mitochondrial morphology in parental (**left** panels) and sorafenib-resistant (**right** panels) Huh7 cells stained with MitoTracker Orange (mitochondria) and DAPI (nuclei) and examined by confocal microscopy. Representative images at 20× magnification. Bar, 10 µm. (**E**) Protein levels of constituents of mitochondrial respiratory complexes in parental, sorafenib-resistant (sorafenib-R) and parental Huh7 cells treated with sorafenib (7.5 µM) for three days (3-day Rx). (**F**) Protein levels of glycolytic enzymes in parental, sorafenib-resistant (sorafenib-R), and parental Huh7 cells treated with sorafenib (7.5 µM for three days; 3-day Rx). (**G**) Expression of mitochondrial complex proteins and glycolytic enzymes in parental and sorafenib-resistant colonies isolated after 3-week continuous exposure to sorafenib.

To verify this hypothesis, we examined the state of mitochondria in parental and resistant Huh7 cells. Sorafenib-resistant cells displayed significantly higher spare respiratory capacity (SRC) along with increased basal oxygen consumption rates (OCR) compared to parental Huh7 cells (Figure 3A). Based on flow cytometry assays, sorafenib-resistant cells exhibited higher mitochondrial membrane potential (Figure 3B) and mitochondrial mass (Figure 3C). Conversely, we observed only modest changes in the extracellular acidification rate (ECAR) and glucose uptake capacity than parental cells (Supplementary Figure S5A,B). Consistent with enhanced mitochondrial biogenesis, resistant cells exhibited an extended mitochondrial network than parental cells (Figure 3D). In sorafenib-resistant cells, individual mitochondria appeared more elongated and tubular than the small rounded mitochondria of parental Huh7 cells, indicating a shift to a hyper-fused phenotype during the adaptive process to chronic exposure to sorafenib. These mitochondrial changes were in contrast with the results of acute exposure to sorafenib. Short-term (6-h or 4-day) treatment with sorafenib had the opposite effect, reducing basal respiratory capacity in parental cells (Supplementary Figure S6A,B).

Thus, whereas acute treatment negatively affected mitochondria, prolonged adaptation to sorafenib was associated with an extensive reorganization of the mitochondrial network, in line with increased expression of mitochondrial protein translation genes. Accordingly, short (3-day) treatment with sorafenib reduced the level of mitochondrial proteins (i.e., NDUFAF1, SDHA, and COX5B) in parental Huh7 cells (Figure 3E and File S1). Conversely, the expression of these mitochondrial proteins recovered, or even increased, in sorafenib-resistant cells. In parallel, the level of glycolytic enzymes (i.e., GAPDH, PKM2, and LDHA) was slightly reduced or unchanged in sorafenib-resistant cells (Figure 3F and File S1). Increased mitochondrial ETC proteins (i.e., NDUFAF1, UQCRQ, COX5B), along with decreased glycolytic enzymes, was also evident in sorafenib-resistant cells isolated under clonal growth condition (Figure 3G and File S1). Therefore, chronic exposure to sorafenib induces an adaptive process that involves enhanced mitochondrial biogenesis.

### 3.5. Genetic and Pharmacological Inhibition of STAT3 Sensitizes Resistant Cells to Sorafenib

**Figure 4:**
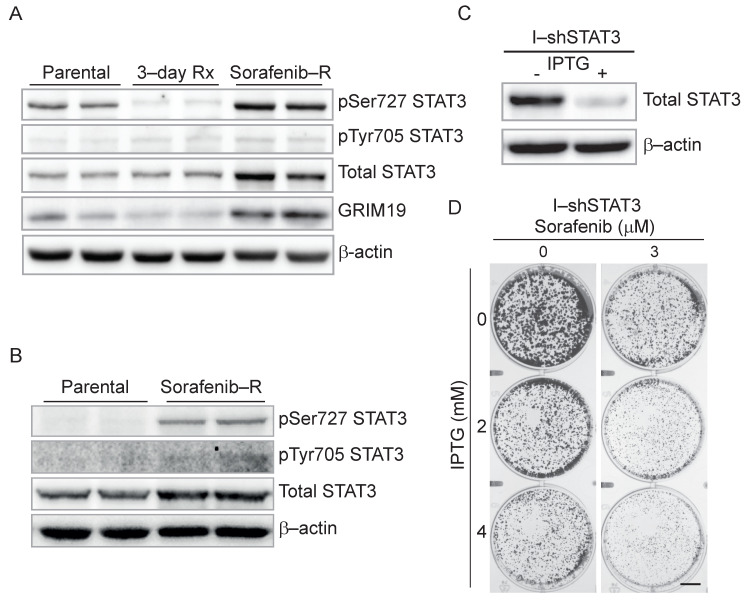
pSer727 STAT3 activation in sorafenib-resistant Huh7 cells. (**A**) Total and phosphorylated STAT3 and GRIM19 levels in parental, sorafenib-treated (3-day Rx) and sorafenib-resistant (sorafenib-R) Huh7 cells determined by western blot. B-actin is a protein loading control. (**B**) Total and phosphorylated STAT3 in parental and sorafenib-resistant Huh7 colonies isolated by 3-week exposure to sorafenib. (**C**) Inducible STAT3 knockdown in sorafenib-resistant Huh7 cells expressing IPTG-inducible short hairpin RNA (shRNA) targeting STAT3 (I-shSTAT3). (**D**) Growth of sorafenib-resistant Huh7 cells exposed to sorafenib with and without induction of STAT3 knockdown by IPTG in clonal growth conditions. Bar: 10 mm.

Transcriptional and mitochondrial reprogramming characterized the emergence of sorafenib-resistant HCC cells. To identify potentially actionable targets for the reversal of sorafenib resistance, we looked at the expression and activation of STAT3, a transcription factor with both nuclear and mitochondrial functions [25]. STAT3 activation by Tyr705 phosphorylation was previously associated with resistance to multiple TKIs and was suggested as a promising target to counteract drug resistance [26]. Accordingly, we examined STAT3 levels in parental and sorafenib-resistant Huh7 cells. Total STAT3 increased in resistant cells, but the level pTyr705 did not change substantially (Figure 4A and File S1). Conversely, pSer727 STAT3 decreased in Huh7 cells after a short treatment with sorafenib and markedly increased in sorafenib-resistant cells. This finding is particularly relevant because mitochondrial STAT3 is phosphorylated at Ser727 and promotes mitochondrial activity [25,27,28]. Concurrent to the change in pSer727 STAT3, sorafenib-resistant cells exhibited increased GRIM19/NDUFA13 (Figure 4A and File S1), a mitochondrial STAT3-interacting protein and a component of the ETC complex I [35]. pSer727 and total STAT3 also increased markedly in sorafenib-resistant Huh7 cells isolated after 3-weeks of continuous treatment in clonal growth conditions (Figure 4B and File S1). Thus, Ser727 phosphorylation of STAT3 occurred rapidly and consistently during the adaptive process to chronic exposure to sorafenib.

This finding pointed to the involvement of pSer727 and mitochondrial STAT3 in sorafenib resistance. Interestingly, STAT3 mRNA was slightly reduced in sorafenib-resistant Huh7 cells (Supplementary dataset S2), suggesting that upregulation of pSer727 and total STAT3 was due to a post-transcriptional mechanism. Furthermore, increased pSer727 and total STAT3 did not activate canonical STAT3 targets, as shown by the minimal overlap between the genes modulated in sorafenib-resistant cells and those regulated by STAT3 and the IL6/JAK/STAT3 pathway (Supplementary Figure S7).

To verify the relevance of STAT3, we knocked down STAT3 with an inducible short hairpin (shRNA) lentiviral construct. The inducible system avoided interference of STAT3 knockdown during the selection process. IPTG treatment of the inducible-shSTAT3 (I-shSTAT3) Huh7 cells depleted cells of STAT3 (Figure 4C and File S1). STAT3 knockdown in sorafenib-resistant Huh7 cells inhibited colony formation and increased the response to sorafenib (Figure 4D and Supplementary Figure S8A). STAT3 depletion reduced slightly mitochondrial membrane potential (Supplementary Figure S8B) but did not affect mitochondrial mass and OCR (Supplementary Figure S8C,D). Thus, transient knockdown of STAT3 enhanced the response to sorafenib, but was unable to fully rescue the mitochondrial phenotype of sorafenib-resistant cells.

**Figure 5:**
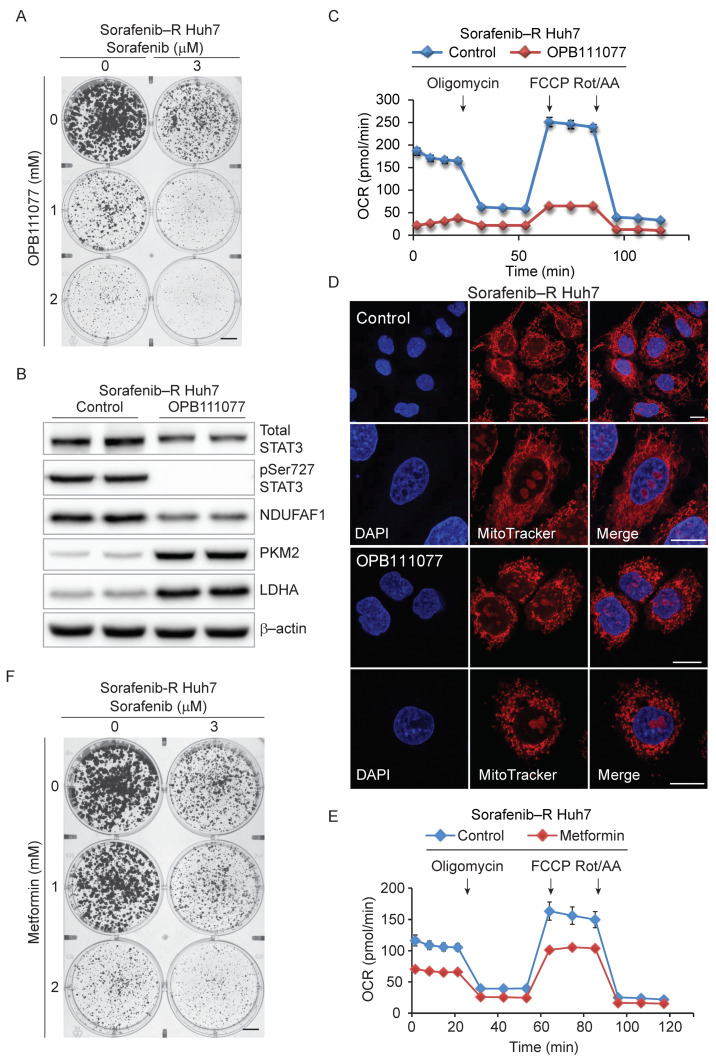
STAT3 inhibition restores mitochondria function and sensitivity to sorafenib. (**A**) Clonal growth of sorafenib-resistant cells treated with sorafenib and increasing concentrations of OPB-111077. (**B**) Expression of total and pSer 727 STAT3, NDUFAF1, PKM2, and LDHA in sorafenib-resistant cells untreated (Control) and treated with OPB-111077 (10 μM, 72 h). (**C**) Mitochondrial OCR in sorafenib-resistant Huh7 cells untreated (Control) and treated with OPB111077 (10 µM, 5 h). Basal respiratory capacity, *p* ≤ 0.005. (**D**) Confocal microscopy images of sorafenib-resistant Huh7 cells untreated (**top** panels) and treated (**bottom** panels) with OPB-111077 (10 µM, 16 h) and stained with Mitotracker Orange and DAPI. Representative images at 10× and 20× magnification. Bars: 10 µm. (**E**) Mitochondrial OCR in sorafenib-resistant Huh7 cells untreated (Control) and treated with metformin (3.5 mM, 5 h). Basal respiratory capacity, *p* ≤ 0.005. (**F**) Clonal growth of sorafenib-resistant cells treated with sorafenib and increasing concentrations of metformin.

We described recently potent small-molecule inhibitors that bind tightly to the SH2 domain of STAT3 and block both nuclear and mitochondrial functions of STAT3 [36,37]. OPB-111077 is a novel derivative that shares the mechanism of action of the parent compounds OPB-51602 and OPB-31121. OPB-111077 has good pharmacokinetics properties and low toxicity and showed promising anticancer activity in phase I–II trials in patients with advanced cancer including HCC [38,39]. We tested whether OPB-111077 could improve the response of resistant cells to sorafenib. Similar to the genetic depletion of STAT3, OPB-111077 inhibited clonal growth of sorafenib-resistant Huh7 cells and, in combination with sorafenib, suppressed colony formation (Figure 5A and Supplementary Figure S9A, File S1).

Notably, OPB-111077 blocked Ser727 phosphorylation of STAT3 in sorafenib-resistant cells (Figure 5B and File S1). Furthermore, OPB-111077 reduced the mitochondrial protein NDUFAF1, whereas it strongly induced PKM2 and LDHA. Using the Seahorse analyzer, we observed a drastic reduction in OCR after OPB-111077 (Figure 5C). Treatment with OPB-111077 also disrupted the enlarged mitochondrial network of resistant cells and restored the pattern of small, rounded mitochondria of parental Huh7 cells (Figure 5D). Thus, OPB-111077 inhibited pSer727 STAT3, reverted the mitochondrial phenotype, and restored the sensitivity of resistant cells to sorafenib. We further explored the concept of the therapeutic targeting of mitochondria-related processes and STAT3 by testing metformin, an antidiabetic drug and an inhibitor of ETC complex I [40]. Metformin reduced OCR in sorafenib-resistant cells (Figure 5E) and inhibited colony formation alone and combined with sorafenib (Figure 5F and Supplementary Figure S9B, File S1), although it was less effective than OPB-111077. These results collectively support the notion of a role of mitochondria reprogramming in sorafenib-resistance and pointed to STAT3 as a relevant player and actionable target, offering new options for the treatment of advanced HCC.

## 4. Discussion

Sorafenib is one of the few approved systemic therapy for advanced-stage HCC [3]. However, the clinical benefits are modest [3]. Many patients are intrinsically refractory or rapidly become resistant to the drug through various poorly defined mechanisms [3,8]. Understanding the biological basis of resistance to TKIs such as sorafenib can inform on means to predict the likelihood of response, overcome resistance, and improve treatment efficacy. Here, we show that human HCC cells adapt to continuous and prolonged exposure to sorafenib and undergo complex reprogramming of the transcriptome and reorganization of the mitochondrial network. At the transcriptional level, this adaptive process implies activating multiple genes involved in critical aspects of mitochondrial biogenesis from mitochondrial protein translation to the mitochondrial import of nuclear-encoded proteins. A highly relevant finding of the study is that the expression of MRP genes identified distinct subgroups of primary HCCs and higher expression marked patients that responded poorly to sorafenib treatment. Thus, both the clinical and in vitro data imply that enhanced ribosome assembly and protein translation in the mitochondria affect the response to sorafenib. We found that counteracting mitochondrial reprogramming by inhibiting STAT3 restored the sensitivity of resistant HCC cells to sorafenib. These data indicate that mitochondrial plasticity and reorganization of the mitochondrial network contribute to drug resistance. Targeting these processes might represent a valid strategy to increase treatment efficacy in HCC patients.

Acute treatment with sorafenib reduced mitochondrial function in HCC cells. However, cells surviving prolonged exposure to sorafenib adapted and exhibited profound changes in mitochondria with increased respiratory capacity and an expanded network of hyper-fused mitochondria. Thus, continuous treatment with sorafenib promotes an adaptive response causing HCC cells to switch to a prevalently mitochondrial phenotype. In addition to increased OXPHOS, this adaptive process could involve additional and more complex changes in mitochondrial metabolism including fatty acid oxidation, contributing to the resistant phenotype [41]. This mitochondrial adaptive response could represent a shared mechanism of innate and acquired resistance of HCCs to many cancer treatments including targeted agents, cytotoxic drugs, and immune therapeutics. Enhanced MPT and reprogramming of mitochondrial functions could be a mechanism of adaptation to environmental challenges and anticancer treatment shared by many tumor types. Despite the notion that cancer cells cover their energy needs substantially through aerobic glycolysis (the so-called Warburg effect), tumor development also relies on functional mitochondria and the ability to reprogram metabolism and mitochondrial activity [15,42]. Indeed, the critical role of mitochondria in tumor progression and response to activation of oncogenic pathways is becoming increasingly evident [43,44]. On the other hand, the dependence on mitochondrial biogenesis and respiration of resistant tumor cells could increase vulnerability to mitochondria-targeting drugs [22,23,43,44].

The relation between tumor metabolism and the therapeutic response to anticancer drugs is the subject of numerous studies. Metabolic plasticity may contribute to sorafenib resistance in HCC [41,45,46]. However, an unequivocal view of the role of energy metabolism and mitochondrial function is still missing [41]. Our present study shows an expansion of the mitochondrial network and a shift toward increased mitochondrial respiration in Huh7 cells surviving prolonged treatment with sorafenib. The mitochondrial phenotypic changes were associated with the upregulation of genes for mitochondrial ribosome assembly, protein translation, and the formation of respiratory complexes. CHCH domain (CHCHCD)-containing proteins CHCHD10, CHCHD4, and CHCHD5, which are involved in protein import, assembly of ETC complexes, and cristae remodeling in mitochondria [33], were overexpressed in resistant Huh7 cells. Notably, we found that sorafenib-resistant cells exhibited significantly higher expression of multiple MRP encoding genes. MRPs constitute the large and small subunits of the mitochondrial ribosomes. The primary function of the mitochondrial ribosomes is to translate the 13 subunits of the respiratory complexes encoded by the mitochondrial DNA [32]. This process is critical for the formation of functional mitochondria. Therefore, the proper supply of MRPs is crucial for ensuring the coordinated production of mitochondrial- and nuclear-encoded components of the mitochondria [32]. An imbalance between the nuclear and mitochondrial processes would be detrimental to cell survival. Thus, enhanced synthesis of MRPs and ribosomal assembly could promote mitochondrial biogenesis and protect from drug-induced metabolic stress and death.

The role of MRPs and MPT in carcinogenesis is an emerging area of investigation. Increased MPT is associated with genetic loss of the tumor suppressors RB1 and TP53 and drives the aggressive phenotype of triple-negative breast cancers promoting mitochondrial OXPHOS, mesenchymal, and stem-like features [47]. K-Ras-driven carcinogenesis also depends heavily on enhanced mitochondria function, a process that interestingly involves mitochondrial STAT3 [27]. This mitochondrial dependency relies on increased MPT, which is crucial for the viability of K-Ras transformed cells [48]. MRPS22 interacts with mitochondrial topoisomerase IB and increases MPT and tumorigenesis in liver and colon cancer models [49]. Deacetylated MRPS5 accumulates in mitochondria, promotes MPT, complex I activation, and production of NAD+, and is critical for maintaining stem-like features in liver cancer cells [50]. We found several MRPs upregulated in primary HCCs from two independent patient cohorts, and their expression marked distinct clusters of primary HCCs. This finding was novel and unexpected, with potential implications for identifying HCC patients with reduced responsiveness to TKI therapy. In our study, higher expression of MRP genes indicated poor survival in sorafenib-treated patients with a minor impact on the total patient population, thus confirming the relationship between MRP expression and response to sorafenib. It would be interesting to verify whether a similar association holds for other TKIs and anticancer therapies [3].

MRPs could promote mitochondrial biogenesis and enhance mitochondrial metabolism, allowing tumor cells to better cope with treatment-induced stress. However, coordinating the import of nuclear-encoded proteins with the translation of mitochondrial-encoded components of the ETC complexes is central to avoiding accumulating unassembled protein subunits in the cytoplasm and mitochondria [51]. Indeed, increased MRPs and MPT could also be a source of mitochondrial stress. Mitochondrial-nuclear protein imbalance can activate the mitochondrial unfolded protein response [51]. In this context, reducing expression of MRPs such as MRPS5 induces a protective response that promotes longevity at the organismal and cellular level and prevents aging [52]. Conversely, mitochondrial-nuclear protein imbalance could also be detrimental by promoting aggressive phenotypes in cancer cells [51]. Thus, alongside controlling the functionality of the mitochondrial respiratory chain, deregulated expression of MRPs could produce pro-survival and anti-aging signals in cancer cells and contribute to treatment resistance and tumor progression.

We sought to address what drives the mitochondrial adaptive process associated with sorafenib resistance. This information could give valuable hints to identify novel targets and design effective therapeutic strategies. Applying TF analysis to the set of genes upregulated in sorafenib-resistant cells, we found many genes that were putative c-MYC targets. This result was consistent with the known role of this TF in controlling many nuclear-encoded mitochondrial proteins. Thus, c-MYC could be a significant driver of mitochondrial reprogramming in resistant cells. E2F1 and YY1 were also associated with upregulated genes in sorafenib-resistant cells. Both these TFs also regulate the expression of nuclear-encoded mitochondrial genes. Loss of RB1 activates transcription of mitochondrial genes through E2F1 [47]. YY1 cooperates with PGC-1α to activate the transcription of mitochondrial genes in an mTOR-dependent manner [53]. In addition to these known regulators of mitochondrial gene transcription, we found that total and pSer727 STAT3 increased markedly in sorafenib-resistant Huh7 cells. The upregulation of pSer727 STAT3 was an intriguing finding since Ser727 phosphorylation preferentially marks mitochondrial STAT3 [25,27,28]. Short-term treatment with sorafenib reduced pSer727 STAT3 concomitant with decreased mitochondrial function. Sorafenib-resistant Huh7 cells instead exhibited higher pSer727 STAT3 and increased mitochondrial activity. Genetic depletion or inhibition of STAT3 with OPB-111077 increased the sensitivity of resistant cells to sorafenib. The STAT3 inhibitor also blocked pSer727 and reverted the mitochondrial phenotypic changes. OPB-111077 belongs to a novel class of inhibitors that affect both the nuclear and mitochondrial function of STAT3 [37], and recent clinical trials have tested it as a single agent in HCC patients [38,39]. Thus, STAT3 inhibition may represent a clinically actionable strategy to overcome innate and acquired resistance to sorafenib. STAT3 could contribute to the resistant phenotype at various levels. STAT3 localizes in mitochondria, is integrated into ETC complex I, and positively affects mitochondrial respiration [25,27,28]. Increased STAT3 could boost mitochondrial activity and promote the phenotypic changes associated with sorafenib resistance. In addition to nuclear-encoded genes, STAT3 can also control transcription of the mitochondrial genome [54]. Although this is a less investigated aspect, mitochondrial STAT3 could contribute to resistance to sorafenib by yet unknown mechanisms. Significantly, various signaling pathways including MAPK kinases could induce pSer727 STAT3 [25]. MAPK12 (p38γ) and MAPK14 (p38α) contribute to liver carcinogenesis and resistance to sorafenib, respectively [9,55]. MAPK12 is involved in mitochondrial biogenesis in a mouse model of skeletal muscle adaptation through the activation of PGC-1α [56]. Thus, activation of p38/MAPK kinases in response to sorafenib-induced stress might contribute to the resistant phenotype in HCC cells by enhancing mitochondrial biogenesis and function through multiple downstream targets, which may include nuclear and mitochondrial STAT3. However, neither MAPK12 nor MAPK14 has known links to mitochondrial reprogramming and STAT3 activation in liver carcinogenesis and drug resistance. Our gene expression analysis showed upregulation of MAPK12, but no change in MAPK14 expression in sorafenib-resistant cells, although their activity might be regulated mainly at the post-transcriptional level. All these aspects, which may have relevant implications for the treatment of HCC, will require further investigation.

## 5. Conclusions

Our study showed a critical involvement of mitochondrial protein translation and plasticity in intrinsic and acquired resistance to sorafenib. These findings, sustained by preclinical data in human cell lines and evidence from HCC patients, suggest that targeting the factors underpinning mitochondrial reprogramming is a good strategy for improving treatment efficacy and clinical outcome in HCC patients.

## Figures and Tables

**Table 1:** Reactome pathway analysis in sorafenib-resistant Huh7 cells.

Index	Name	*p*-Value	Adjusted *p*-Value ^3^	Z-Score	Combined Score
Upregulated Genes ^1^					
1	rRNA processing	2.2 × 10^–6^	1.3 × 10^–3^	−2.03	26.39
2	Mitochondrial translation elongation	5.0 × 10^–6^	1.3 × 10^–3^	−2.07	25.26
3	Mitochondrial translation termination	5.0 × 10^–6^	1.3 × 10^–3^	−2.06	25.18
4	Mitochondrial translation initiation	5.0 × 10^–6^	1.3 × 10^–3^	−2.04	24.84
5	Major pathway of rRNA processing in the nucleolus	6.4 × 10^–6^	1.3 × 10^–3^	−2.01	24.07
6	Mitochondrial translation	1.2 × 10^–5^	2.0 × 10^–3^	−2.07	23.39
**Downregulated Genes ^2^**					
1	Platelet degranulation	1.1 × 10^–6^	1.0 × 10^–3^	−1.92	26.47
2	Response to elevated platelet cytosolic Ca^2+^	2.1 × 10^–6^	1.1 × 10^–3^	−1.90	24.77
3	Biological oxidations	1.2 × 10^–5^	2.4 × 10^–3^	−2.02	22.86
4	Phase II conjugation	1.0 × 10^–5^	2.4 × 10^–3^	−1.91	21.95
5	Amyloid fiber formation	9.6 × 10^–6^	2.4 × 10^–3^	−1.89	21.80

^1^ Genes upregulated in sorafenib-resistant Huh7 cells compared to parental huh7 cells. ^2^ Genes downregulated in sorafenib-resistant cells compared to parental Huh7 cells. ^3^ Adjusted *p*-value by the Benjamini–Hochberg method.

**Table 2:** Transcription factor enrichment analysis in sorafenib-resistant Huh7 cells.

Index	Name	*p*-Value	Adjusted *p*-Value ^3^	Z-Score	Combined Score
Upregulated Genes ^1^					
1	MYC_ENCODE	1.7 × 10^–13^	1.7 × 10^–11^	−1.62	47.63
2	MAX_ENCODE	3.9 × 10^–9^	2.0 × 10^–7^	−1.60	31.06
3	MYC_CHEA	5.9 × 10^–8^	2.0 × 10^–6^	−1.66	27.66
4	ZBTB7A_ENCODE	2.2 × 10^–6^	5.8 × 10^–5^	−1.55	20.16
5	KAT2A_ENCODE	5.2 × 10^–5^	1.1 × 10^–3^	−1.67	16.46
**Downregulated Genes ^2^**					
1	SOX2_CHEA	3.0 × 10^–7^	3.1 × 10^–5^	−1.75	26.37
2	NFE2L2_CHEA	2.6 × 10^–6^	1.2 × 10^–4^	−1.60	20.82
3	UBTF_ENCODE	3.8 × 10^–5^	1.3 × 10^–3^	−1.60	16.29
4	ESR1_CHEA	7.1 × 10^–5^	1.7 × 10^–3^	−1.67	15.99
5	FOSL2_ENCODE	8.4 × 10^–5^	1.7 × 10^–3^	−1.63	15.33
6	TRIM28_CHEA	2.3 × 10^–4^	4.0 × 10^–3^	−1.56	13.04

^1^ Genes upregulated in sorafenib-resistant Huh7 cells compared to parental huh7 cells. ^2^ Genes downregulated in sorafenib-resistant cells compared to parental Huh7 cells. ^3^ Adjusted *p*-value by the Benjamini–Hochberg method.

## Data Availability

Gene expression data from human cell lines generated in this study are publicly available through the Gene Expression Omnibus (GEO) data repository. The data can be found at https://www.ncbi.nlm.nih.gov/geo/ accession number GSE189711.

## Supplementary Materials

The following are available online: https://www.mdpi.com/article/10.3390/cancers13236029/s1. Supplementary Materials and Methods; Supplementary File S1: original images; Supplementary Figure S1: Response of human HCC cell lines to sorafenib; Supplementary Figure S2: Growth properties of parental and sorafenib resistant cells; Supplementary Figure S3: MRP expression in HCC patients; Supplementary Figure S4: Mitochondrial ribosomal proteins and clinical outcome in HCC; Supplementary Figure S5: Glycolytic metabolism in parental and sorafenib resistant Huh7 cells; Supplementary Figure S6: Mitochondrial metabolism in sorafenib-treated Huh7 cells; Supplementary Figure S7: JAK/STAT3 pathway in sorafenib-resistant cells; Supplementary Figure S8: Impact of STAT3 depletion on sorafenib resistance; Supplementary Figure S9: Effects of STAT3 inhibition on sorafenib resistance. Supplementary Table S1: Gene Ontology (GO) enrichment analysis of cellular components; Supplementary Table S2: Putative Myc/Max targets among genes upregulated in sorafenib-resistant cells; Supplementary Table S3: Putative SOX2 targets among genes downregulated in sorafenib-resistant cells. Supplementary Table S4. Gene set enrichment analysis of drug perturbations in sorafenib-resistant cells. Supplementary dataset S1: Upregulated genes in sorafenib-resistant Huh7 cells. Supplementary dataset S2: Downregulated genes in sorafenib-resistant Huh7 cells.
