# Supplementary material for: Mitochondrial Plasticity Promotes Resistance to Sorafenib and Vulnerability to STAT3 Inhibition in Human Hepatocellular Carcinoma
Source: Cancers (Basel). 2021 Nov 30;13(23):6029. doi: 10.3390/cancers13236029 (PMC8657239; doi:10.3390/cancers13236029)
Supplement: Supplementary file 1 [file cancers-13-06029-s001.zip › Supplementary figure S1-b JM.pdf]

A

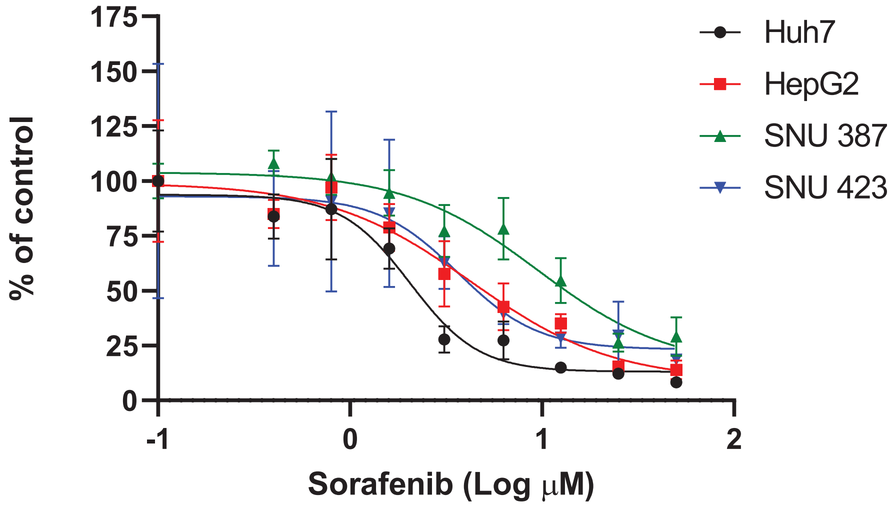

B

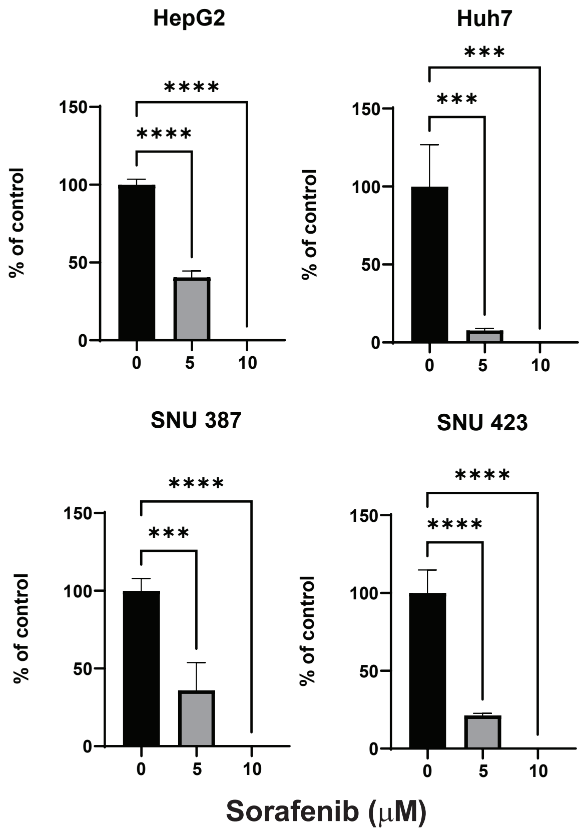

Supplementary figure S1. Response of human HCC cell lines to sorafenib. (A) Proliferation of human HCC cell lines incubated with sorafenib. Cell viability was measured after 72 h by the MTT assay. (B) Growth of HepG2, Huh7, SNU-387, and SNU-423 exposed to sorafenib in colony-forming assays.
