# Supplementary material for: Mitochondrial Plasticity Promotes Resistance to Sorafenib and Vulnerability to STAT3 Inhibition in Human Hepatocellular Carcinoma
Source: Cancers (Basel). 2021 Nov 30;13(23):6029. doi: 10.3390/cancers13236029 (PMC8657239; doi:10.3390/cancers13236029)
Supplement: Supplementary file 1 [file cancers-13-06029-s001.zip › Supplementary figure S2-b.pdf]

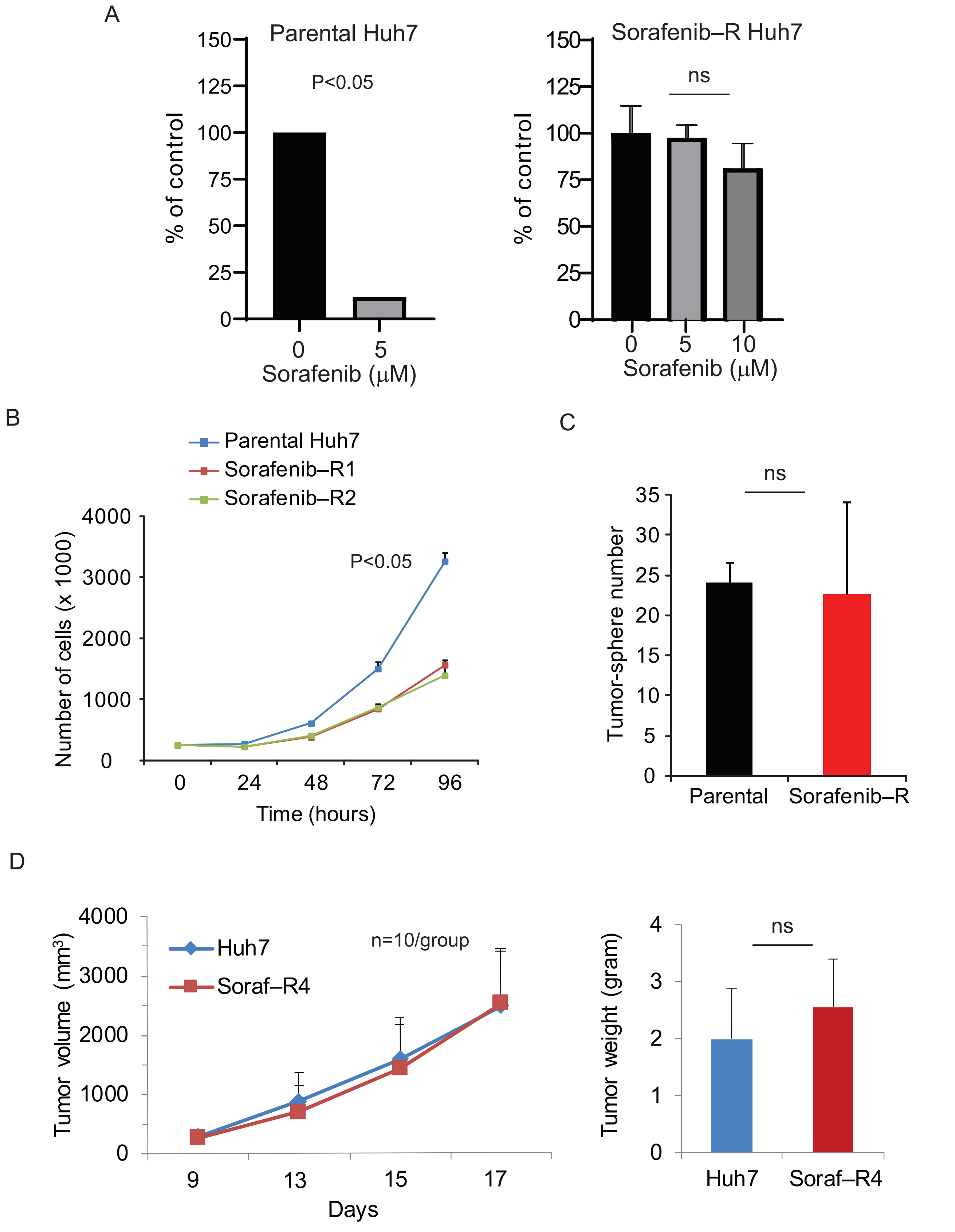


Supplementary figure S2. Growth properties of parental and sorafenib resistant cells. (A) Quantification of colony formation by parental (left panel) and sorafenib-resistant (right panel) Huh7 cells exposed to sorafenib shown in Figure 1B. (B) Proliferation of parental and sorafenib-resistant (Sorafenib-R1 and R2) Huh7 cells in adherent cell culture conditions determined by cell counting. (C) Tumor-sphere forming ability of parental and sorafenib-resistant Huh7 cells in 3D stem cell-selective culture conditions. (D) Growth of subcutaneous xenografts of parental and sorafenib-resistant Huh7 cells in nude mice (n=10 mice/group). Right panel, mean tumor weight at the end of the experiment.
