# Supplementary material for: Mitochondrial Plasticity Promotes Resistance to Sorafenib and Vulnerability to STAT3 Inhibition in Human Hepatocellular Carcinoma
Source: Cancers (Basel). 2021 Nov 30;13(23):6029. doi: 10.3390/cancers13236029 (PMC8657239; doi:10.3390/cancers13236029)
Supplement: Supplementary file 1 [file cancers-13-06029-s001.zip › Supplementary figure S3-b.pdf]

A

All patients						
	Stage I	Stage II	Stage IIIA	Stage IIIB-C	Stage IV	Total
Cluster 1	100	39	40	12	2	193
Cluster 2	51	32	24	3	1	111
Cluster 3	19	12	2	2	1	36
Total	170	83	66	17	4	340

Sorafenib-treated patients						
	Stage I	Stage II	Stage IIIA	Stage IIIB-C	Stage IV	Total
Cluster 1	6	4	6	3	1	20
Cluster 2	1	2	1	1	0	5
Cluster 3	1	1	0	0	0	2
Total	8	7	7	4	1	27

B

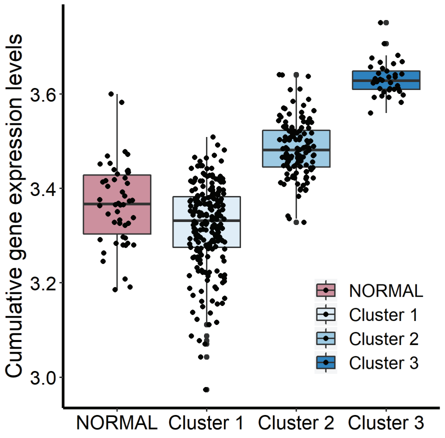

	NORMAL	Cluster 1	Cluster 2
Cluster 1	3.61×10^{-3}	-	-
Cluster 2	1.09×10^{-14}	7.43×10^{-42}	-
Cluster 3	5.02×10^{-15}	8.39×10^{-22}	3.70×10^{-18}

C

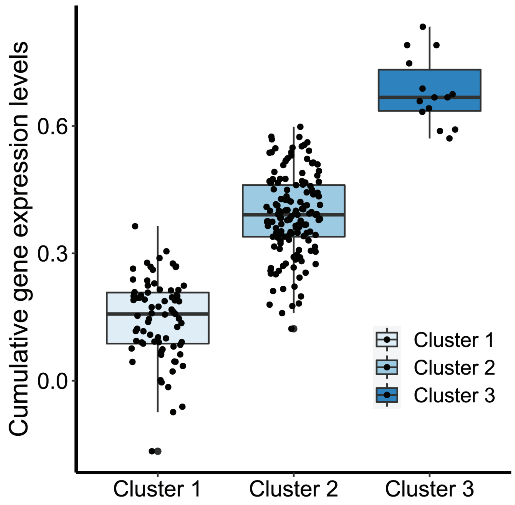

	Cluster 1	Cluster 2
Cluster 2	5.13×10^{-30}	-
Cluster 3	3.15×10^{-09}	1.20×10^{-09}

D

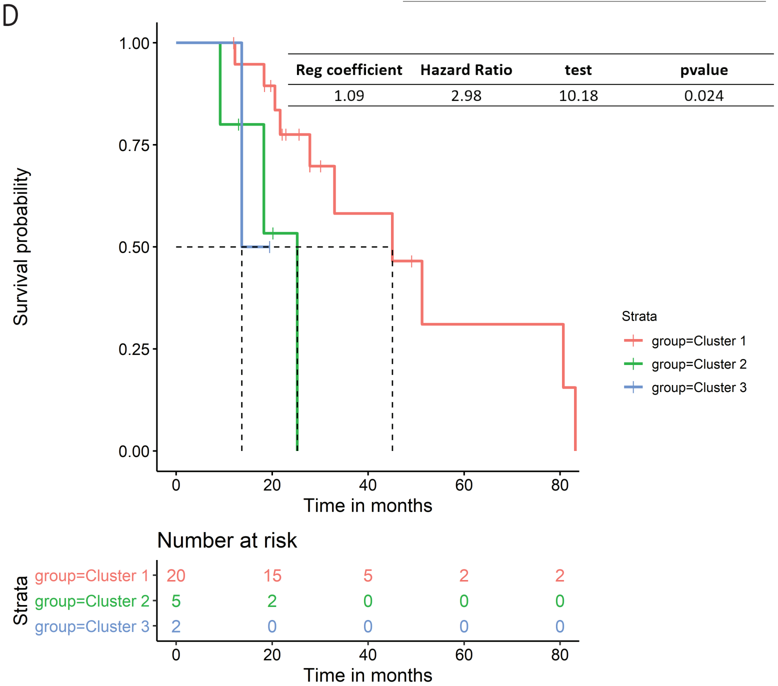

E

Predictor	Reg coefficient	Hazard ratio	*p*-value
Cluster	-0.27	0.76	0.265
Expression value	1.23	3.43	0.295
Stage	0.43	1.53	5.03×10^{-7}

F

Predictor	Reg coefficient	Hazard ratio	*p*-value
Cluster	-0.38	0.68	0.640
Expression value	11.35	84963.16	0.008
Stage	0.58	1.77	0.065

Supplementary figure S3. MRP expression in HCC patients. (A) Distribution according to stage and MRP-based clustering of patients in the total cohort (Top) and in the subset of patients treated with sorafenib (Bottom) in the TCGA dataset. (B) Differential clustering of HCC and normal liver samples in the TCGA cohort and cumulative MRP gene set expression. Total numbers of normal liver and HCC samples are 50 and 340. Bottom, p-values for all class comparisons. (C) Differential clustering of HCC samples in the ICGC patient cohort and cumulative MRP gene set expression. Total number of HCC samples included in the analysis is 240. Bottom, p-values for all class comparisons. (D) MRP-based clusters and overall survival in sorafenib-treated patients in the TCGA cohort. (E-F) Cox regression multivariate survival analysis considering MRP gene expression, clustering and stage in the entire TCGA cohort (E) and the sorafenib-treated patients (F).
