# Supplementary material for: Mitochondrial Plasticity Promotes Resistance to Sorafenib and Vulnerability to STAT3 Inhibition in Human Hepatocellular Carcinoma
Source: Cancers (Basel). 2021 Nov 30;13(23):6029. doi: 10.3390/cancers13236029 (PMC8657239; doi:10.3390/cancers13236029)
Supplement: Supplementary file 1 [file cancers-13-06029-s001.zip › Supplementary figure S4-b.pdf]

A

Gene name	Reg coefficient	Hazard Ratio	Like test	Like test - pvalue
**MRPL17**	2.310766	10.08214	16.89001	0.001228
**MRPS16**	2.708782	15.01098	13.24604	0.004234
**MRPS15**	1.340518	3.821023	10.18299	0.014647
**MRPL27**	1.260259	3.526336	8.896445	0.019095
**MRPS9**	1.986407	7.289293	8.759591	0.019095
**MRPL18**	1.750993	5.76032	8.166346	0.019446
**MRPL35**	2.554495	12.8648	8.013895	0.019446
**MRPL12**	0.970765	2.639963	7.872779	0.019446
**MRPS12**	1.062015	2.892194	7.641021	0.019652
**MRPL58**	1.12367	3.076121	6.879689	0.025799
**MRPS34**	1.135695	3.113336	6.792403	0.025799
**MRPS7**	1.16922	3.219479	6.510251	0.027707
**MRPL55**	0.882749	2.417537	5.734214	0.037273
**MRPL24**	0.81763	2.265125	5.438468	0.037273
**MRPL48**	1.533477	4.634264	5.380914	0.037273
**MRPS30**	1.512466	4.537907	5.318551	0.037273
**MRPL37**	1.211836	3.359647	5.295688	0.037273
**MRPS21**	0.882611	2.417202	5.274312	0.037273
**MRPS23**	1.116869	3.055272	5.019171	0.040901
**MRPL3**	1.909337	6.748616	4.841666	0.043059
**MRPL54**	0.977107	2.656759	4.398719	0.052694
**MRPL33**	1.187661	3.279402	4.332286	0.052694
**MRPS26**	1.032373	2.807722	3.865535	0.066431
**MRPL10**	0.775259	2.171155	3.731865	0.068954
**MRPL11**	0.942414	2.56617	3.195733	0.09155
**MRPL53**	0.747651	2.112034	1.419659	0.278356
**MRPL44**	0.958727	2.608374	1.23849	0.305135
**MRPL38**	0.52409	1.688921	1.16245	0.311061
**MRPL1**	0.415171	1.51463	0.549826	0.490002
**MRPS35**	0.576116	1.779114	0.485237	0.502262
**MRPL42**	0.169699	1.184949	0.041192	0.839168

B

Gene name	Reg coefficient	Hazard Ratio	Like test	Like test - pvalue
**MRPL53**	0.48597	1.625751	7.64365	0.152261
**MRPL3**	0.541106	1.717905	5.647432	0.152261
**MRPL54**	-0.26779	0.765066	5.469733	0.152261
**MRPS23**	0.357904	1.430328	5.443026	0.152261
**MRPL17**	0.297957	1.347103	4.541616	0.205098
**MRPL38**	-0.32412	0.723162	2.592867	0.55462
**MRPL11**	0.175745	1.192133	1.714818	0.639887
**MRPL10**	0.224194	1.251314	1.658535	0.639887
**MRPS15**	0.155275	1.167979	1.538226	0.639887
**MRPL12**	0.114776	1.121622	1.43586	0.639887
**MRPS16**	0.200229	1.221683	1.273495	0.639887
**MRPL37**	0.154165	1.166683	1.235775	0.639887
**MRPL33**	0.143985	1.154867	1.223728	0.639887
**MRPL48**	0.184699	1.202856	1.124363	0.639887
**MRPS7**	0.151477	1.163552	0.963388	0.674425
**MRPS9**	0.163774	1.177948	0.865618	0.682332
**MRPS30**	0.162418	1.176352	0.646724	0.768228
**MRPS21**	0.081494	1.084907	0.523285	0.808487
**MRPL55**	-0.06273	0.939201	0.35763	0.868801
**MRPL1**	0.087459	1.091398	0.33881	0.868801
**MRPL18**	0.084459	1.088128	0.269589	0.89104
**MRPS12**	0.028727	1.029143	0.078829	0.990223
**MRPS26**	0.029759	1.030206	0.049786	0.990223
**MRPL27**	-0.01825	0.981914	0.027372	0.990223
**MRPS35**	-0.02859	0.971816	0.023367	0.990223
**MRPL35**	-0.0284	0.971999	0.019469	0.990223
**MRPL42**	0.035622	1.036264	0.018545	0.990223
**MRPL44**	0.013521	1.013613	0.004871	0.990223
**MRPS34**	-0.00418	0.995828	0.001324	0.990223
**MRPL58**	0.002665	1.002668	0.000329	0.990223
**MRPL24**	0.001269	1.00127	0.00015	0.990223

Supplementary figure S4. Mitochondrial ribosomal proteins and clinical outcome in HCC. Association of the expression of MRP genes and overall survival in the subset of sorafenib-treated HCC patients (A) and in the total HCC patient population (B) in the TCGA cohort by Cox proportional hazard regression analysis. MRP genes aligned by increasing p-value.
