# Supplementary material for: Mitochondrial Plasticity Promotes Resistance to Sorafenib and Vulnerability to STAT3 Inhibition in Human Hepatocellular Carcinoma
Source: Cancers (Basel). 2021 Nov 30;13(23):6029. doi: 10.3390/cancers13236029 (PMC8657239; doi:10.3390/cancers13236029)
Supplement: Supplementary file 1 [file cancers-13-06029-s001.zip › Supplementary figure S5-b.pdf]

A

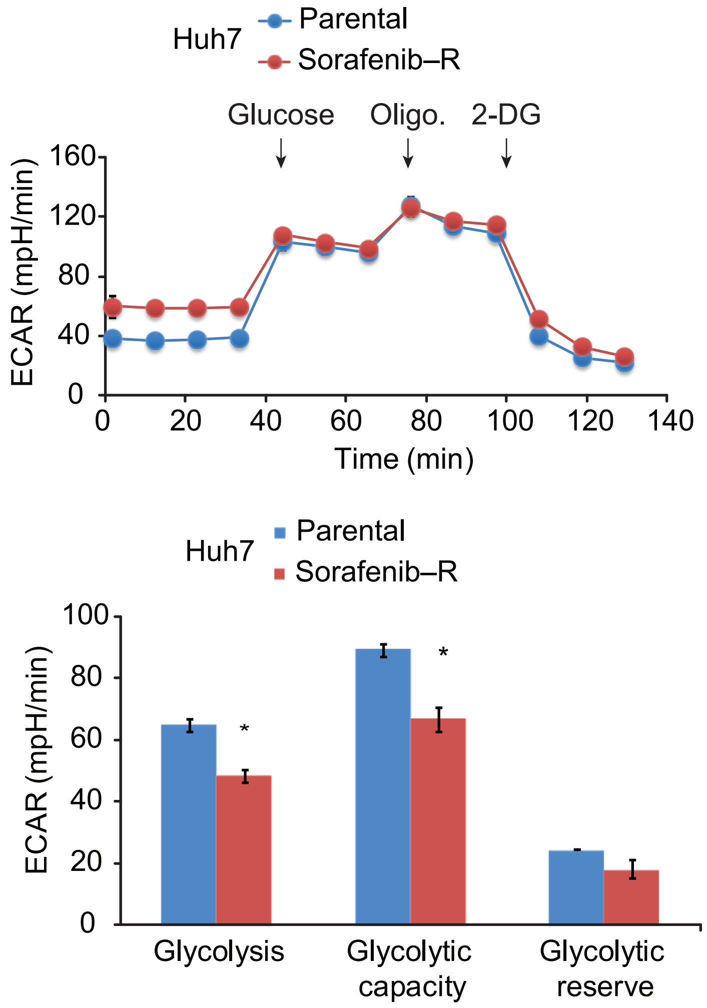


B

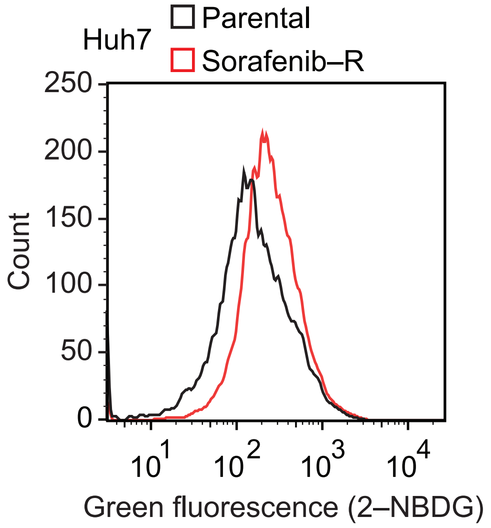


**Supplementary figure S5. Glycolytic metabolism in parental and sorafenib resistant Huh7 cells.** (A) Extracellular acidification rate (ECAR) assessed using Seahorse XFp analyzer in parental and sorafenib-resistant Huh7 cells. Top, real-time OCR measurements normalized to cell number. Bottom, basal and spare respiratory capacity based on OCR profiles. * P ≤ 0.05. (B) Glucose uptake assessed by flow cytometry analysis of 2-NBDG-stained parental and sorafenib-resistant Huh7 cells.
