# Supplementary material for: Mitochondrial Plasticity Promotes Resistance to Sorafenib and Vulnerability to STAT3 Inhibition in Human Hepatocellular Carcinoma
Source: Cancers (Basel). 2021 Nov 30;13(23):6029. doi: 10.3390/cancers13236029 (PMC8657239; doi:10.3390/cancers13236029)
Supplement: Supplementary file 1 [file cancers-13-06029-s001.zip › Supplementary figure S6-b.pdf]

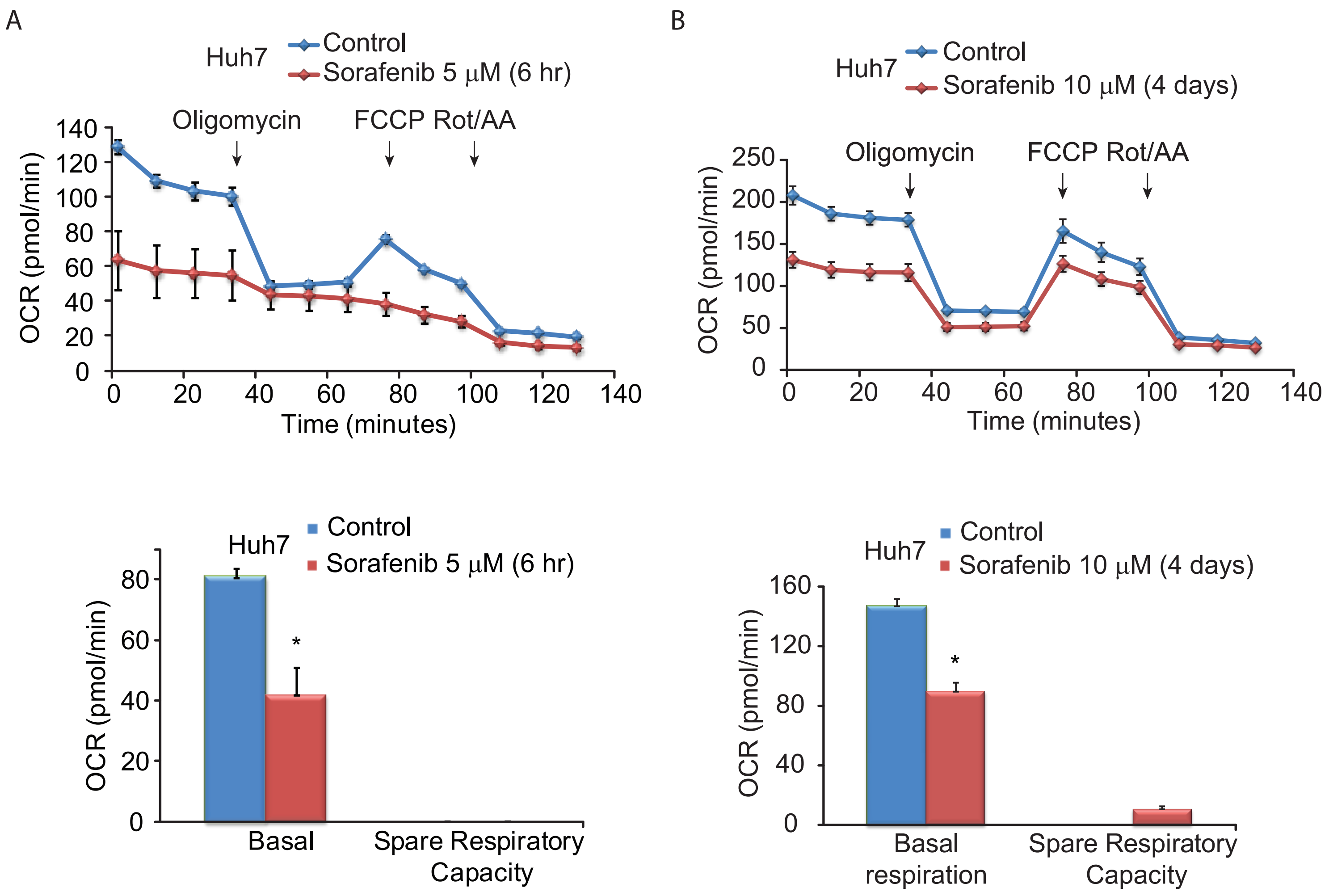

Supplementary figure S6. Mitochondrial metabolism in sorafenib-treated Huh7 cells. (A-B) Mitochondrial OCR in parental Huh7 cells treated with sorafenib (5-10 μM) for 6 h (A) and 4 days (B). *Top panels*, real-time OCR measurements normalized to cell number. *Bottom panels*, basal and spare respiratory capacity based on OCR profiles.
