# Supplementary material for: Mitochondrial Plasticity Promotes Resistance to Sorafenib and Vulnerability to STAT3 Inhibition in Human Hepatocellular Carcinoma
Source: Cancers (Basel). 2021 Nov 30;13(23):6029. doi: 10.3390/cancers13236029 (PMC8657239; doi:10.3390/cancers13236029)
Supplement: Supplementary file 1 [file cancers-13-06029-s001.zip › Supplementary figure S7-b.pdf]

A

Standard name	HALLMARK_IL6_JAK_STAT3_SIGNALING
Systematic name	M5897
Brief description	Genes up-regulated by IL6 [GeneID=3569] via STAT3 [GeneID=6774], e.g., during acute phase response.
Full description or abstract	
Collection	H: hallmark gene sets

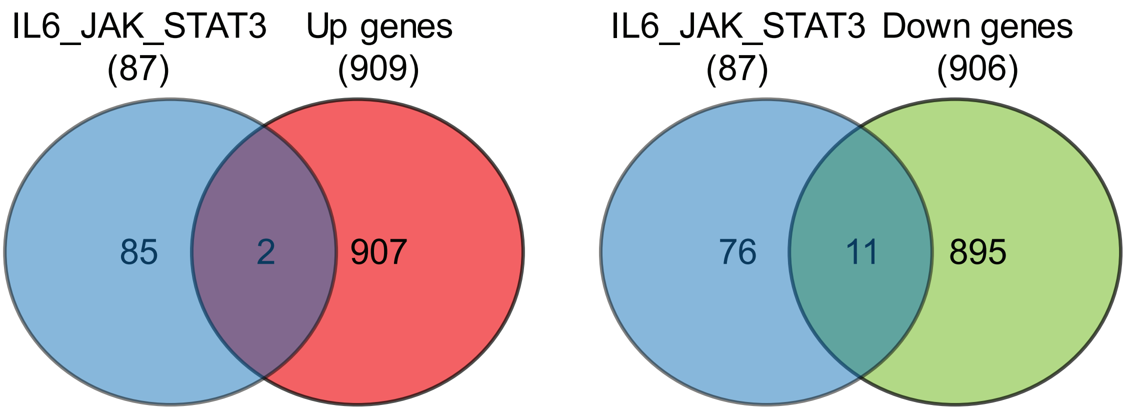

B

Standard name	STAT3_02
Systematic name	M3263
Brief description	Genes having at least one occurence of the transcription factor binding site V$STAT3_02 (v7.4 TRANSFAC) in the regions spanning up to 4 kb around their transcription starting sites.
Full description or abstract	
Collection	C3: motif gene sets<br>TFT: transcription factor targets

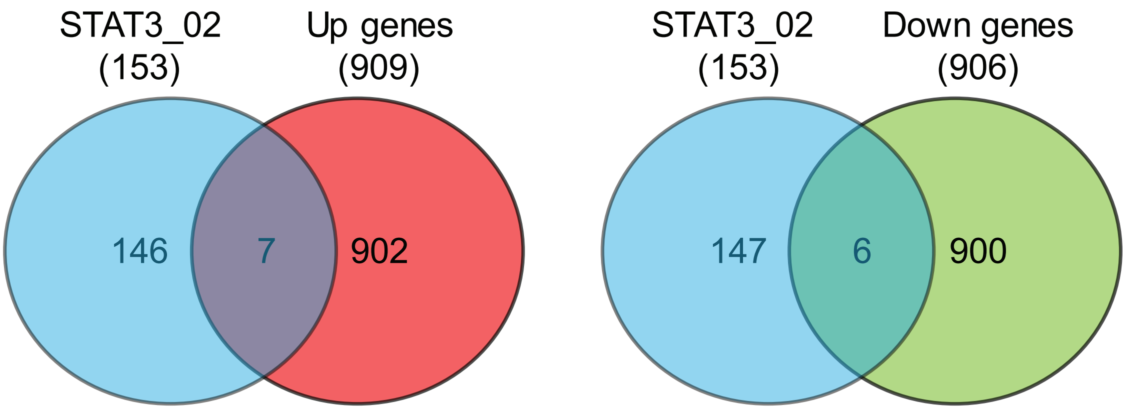

Supplementary figure S7. JAK/STAT3 pathway in sorafenib-resistant cells. (A-B) Presence of IL6/JAK/STAT3 regulated genes (A) and STAT3 target genes (B) among up- and down- regulated genes in sorafenib-resistant cells.
