# Supplementary material for: Mitochondrial Plasticity Promotes Resistance to Sorafenib and Vulnerability to STAT3 Inhibition in Human Hepatocellular Carcinoma
Source: Cancers (Basel). 2021 Nov 30;13(23):6029. doi: 10.3390/cancers13236029 (PMC8657239; doi:10.3390/cancers13236029)
Supplement: Supplementary file 1 [file cancers-13-06029-s001.zip › Supplementary figure S8-b.pdf]

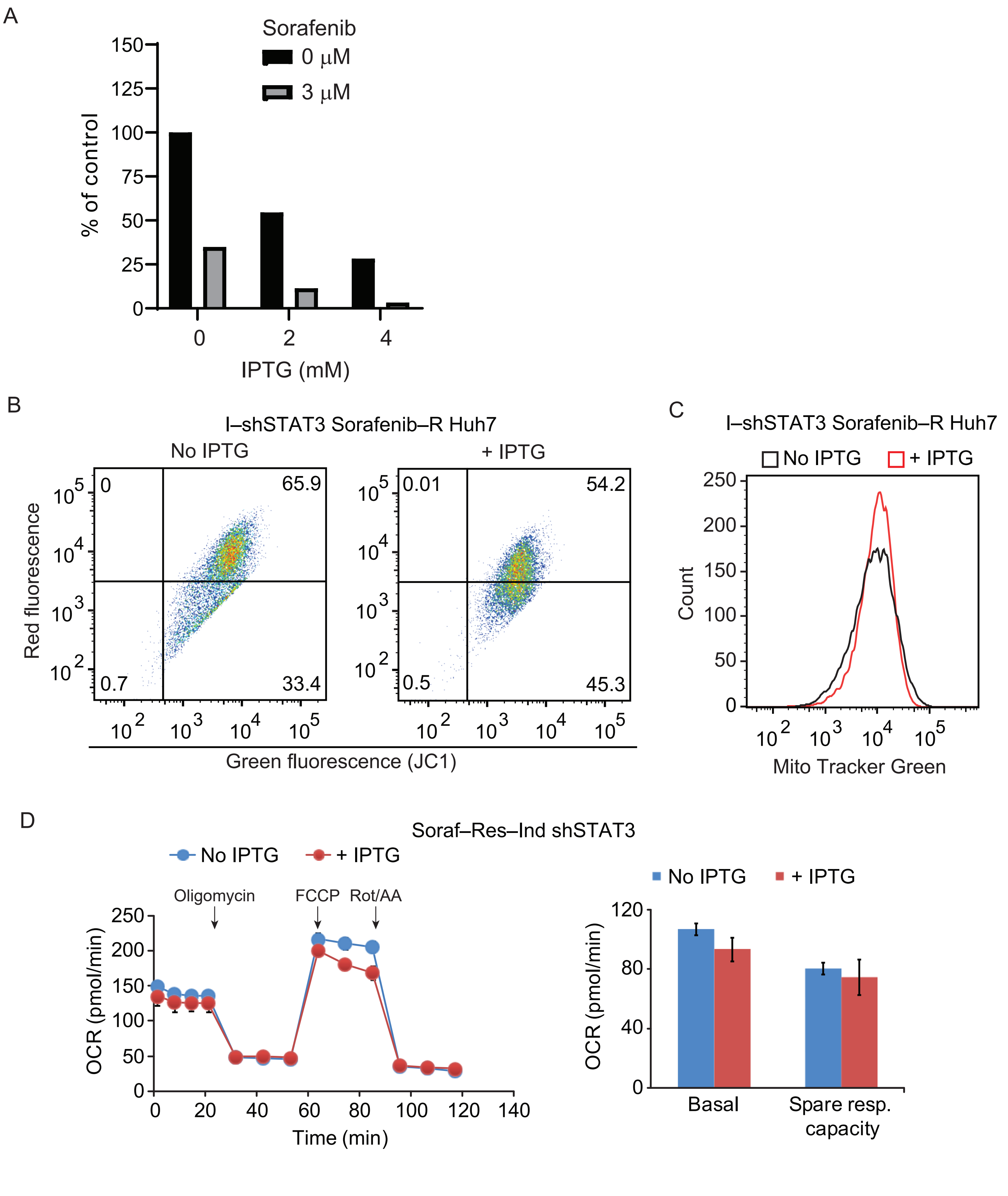

Supplementary figure S8. Impact of STAT3 depletion on sorafenib resistance. (A) Quantification of colony formation by sorafenib-resistant cells treated with sorafenib with and without induction of STAT3 knockdown by IPTG as shown in Figure 3D. (B-C) Mitochondrial membrane potential by JC1 staining (B) and mitochondrial mass by MitoTracker Green staining (C) in sorafenib-resistant Huh7 cells expressing inducible shRNA targeting STAT3. Cells were treated with IPTG (5 mM) for 7 days prior to flow cytometry analysis. (D) Mitochondrial OCR in sorafenib-resistant Huh7 cells with and without inducible STAT3 knockdown. Cells were treated for 7 days with IPTG (5 mM) prior to mitochondrial stress test and OCR measurement by Seahorse XFp analyzer.
