# Supplementary material for: Mitochondrial Plasticity Promotes Resistance to Sorafenib and Vulnerability to STAT3 Inhibition in Human Hepatocellular Carcinoma
Source: Cancers (Basel). 2021 Nov 30;13(23):6029. doi: 10.3390/cancers13236029 (PMC8657239; doi:10.3390/cancers13236029)
Supplement: Supplementary file 1 [file cancers-13-06029-s001.zip › Supplementary figure S9-b.pdf]

A

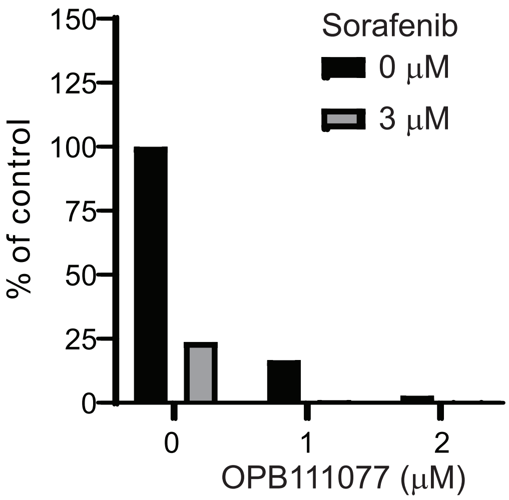

B

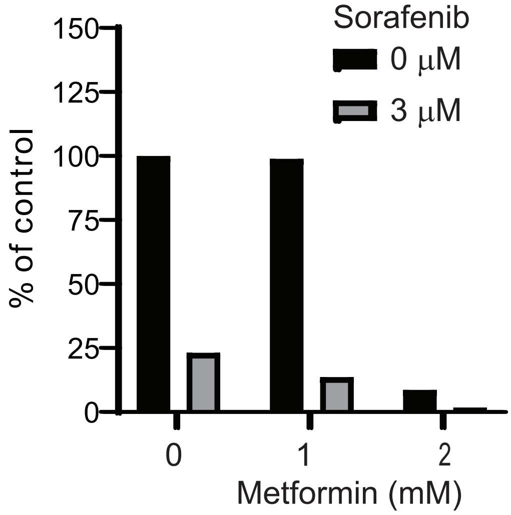

Supplementary figure S9. Effects of STAT3 inhibition on sorafenib resistance. (A) Quantification of colony formation by sorafenib-resistant cells treated with sorafenib and OPB-111077 as shown in Figure 5A. (B) Quantification of colony formation by sorafenib-resistant cells treated with sorafenib and metformin as shown in Figure 5F.
