# Supplementary material for: Mitochondrial Plasticity Promotes Resistance to Sorafenib and Vulnerability to STAT3 Inhibition in Human Hepatocellular Carcinoma
Source: Cancers (Basel). 2021 Nov 30;13(23):6029. doi: 10.3390/cancers13236029 (PMC8657239; doi:10.3390/cancers13236029)
Supplement: Supplementary file 1 [file cancers-13-06029-s001.zip › Supplementary information_Pandit_11_2021.pdf]

Supplementary Information

Mitochondrial plasticity promotes resistance to sorafenib and vulnerability to STAT3 inhibition in human hepatocellular carcinoma

Shusil K. Pandit[¶], Giada Sandrini, Jessica Merulla, Valentina Nobili, Xin Wang[§], Alessia Zangari, Andrea Rinaldi, Dheeraj Shinde, Giuseppina M. Carbone, and Carlo V. Catapano[*]

Tumor Biology and Experimental Therapeutics Program, Institute of Oncology Research, Università della Svizzera Italiana, 6500 Bellinzona, Switzerland.

Supplementary information file include:

- Supplementary materials and methods
- Supplementary figure legends
- Supplementary table legends
- Supplementary dataset

Supplementary Materials and Methods

Cell growth and tumorigenicity. For cell proliferation assays, cells (5 x10^3/well) were seeded in triplicate in 96-well plates and treated with the drug after 24 h. MTT (3-(4, 5-dimethylthiazolyl-2)-2, 5-diphenyltetrazolium bromide) was added after 72 h, followed by the addition of SDS-HCl and reading the absorbance at 570 nm. Colony-forming assays were performed by seeding cells (3-5 x 10^3/well) in 6-well plates in triplicate followed by drug treatment after 24 h [1]. Colonies were stained with 1% crystal violet in 20% ethanol. For tumor-sphere assays, cells (3 x10^3/well) were seeded in 6-well plates coated with poly(2-hydroxyethylmethacrylate) (Poly-HEMA, Sigma-Aldrich) in serum-free medium DMEM-F12 1:1 media (Gibco). Plates were incubated for 8-10 days and examined by light microscopy. For tumor growth in mice, parental and sorafenib resistant Huh7 cells (5 x10^6/mice) were mixed with Matrigel and injected subcutaneously in the flank of athymic female nude mice (4-6 week old, Invigo) [1]. Growth of subcutaneous tumors was monitored by caliper and tumor volume was calculated as: width × length^2)/2 [1]. The protocol for the animal study was approved by Swiss veterinary authority (Cantonal committee for experiments on animals, CCEA) in agreement with national and international guidelines.

Bioinformatics data analysis. Analyses were performed downloading the liver cancer TCGA and ICGC datasets along with their clinical annotations. All bioinformatics analyses were performed in R environment (https://www.R-project.org/). Expression data of the upregulated MRP genes in sorafenib-resistant Huh7 cells were retrieved according to DESeq pipeline [2]. The HCC samples (TCGA, n=371; ICGC, n=240) were clustered with the unsupervised hierarchical clustering (distance = euclidean) technique. The obtained clusters were validated through clValid package

[3], and the final number of clusters was chosen in accordance with clustering quality measures, including Silhouette Width and Average Distance between Means. The Pairwise Wilcoxon Test was carried out in order to establish the significance of the difference between the identified groups. The Gene Set Variation Analysis (GSVA) method, which estimates the relative enrichment of a gene-set of interest across a sample population, allowed representing the expression of the MRP genes as a single cumulative value for each patient. The Enrichment Score (ES) was defined as cumulative MRP gene expression. Then, the ES value was used to test any association with patient survival. The corresponding clinical annotations, including sorafenib treatment, were downloaded using the GDCquery, GDCdownload, GDCprepare functions belonging to the TCGAbiolinks package setting "Clinical Supplement" as data.type and “TCGA-LIHC” as project [4-6]. The parameters of the GDCquery function were set as follows: Project = TCGA-LIHC, Data.category = Clinical, Data.type = Clinical Supplement, Data.format = BCR Biotab. The variable containing the pharmacological information is “clinical_drug_lihc”> pharmaceutical_therapy_drug_name”. The Cox Proportional Hazard regression analysis assessed the effect of several predictor variables (ES, MRP gene expression values, and stage) on the overall survival time simultaneously. The likelihood ratio test, suitable for small sample sizes, was used to establish the significance of the predictive model. The feature selection approach was adopted to identify the MRP genes with greater predictive power. The multiple-test corrected p-values were exploited as the ranking criteria to determine the relevance to individual genes. The HCC samples were divided into two groups according to each gene's median expression value. The Kaplan Meier curves were plotted using the Log Rank Test to compare the data.

Western blot analysis. Cell lysates were prepared using a buffer containing 150 mM NaCl, 1.0% NP40, 0.5% deoxycholate, 0.1% SDS, and 50 mM Tris (pH 8.0) supplemented with Protease and Phosphatase Inhibitor Cocktails (Roche). Protein concentration was measured using the BCA Protein Assay Kit (Pierce). Protein samples (30 µg) were loaded on 10%-15% SDS-polyacrylamide gels for electrophoresis and then transferred to PVDF membranes for immunoblotting with indicated antibodies. Membranes were incubated with the following primary antibodies: STAT3, pTyr705 STAT3, pSer727 STAT3 and β-actin (Cell Signaling); NDUFAF1, SDHA, COX5B, UQCRQ, ATP5G1, PKM2, LDHA and GRIM19 (Abcam); GAPDH (Millipore); and anti-rabbit HRP, anti-mouse HRP (Amersham Biosciences) secondary antibodies. Immunoblots were imaged using Fusion Solo.

Oxygen Consumption Rate (OCR) and Extracellular Acidification Rate (ECAR). OCR were measured using Seahorse XFp Analyzer (Agilent-Seahorse Bioscience) [1]. Cells (10-15 x 10^3) were seeded in miniplates 24 h prior to OCR measurement. Mitochondrial Stress test was performed by pre-incubating cells in the XF base medium supplemented with 1 mM pyruvate, 2 mM glutamine and 10 mM glucose according to manufacturer's protocol. OCR measurements were taken in basal condition and after the addition of oligomycin (1 µM), FCCP (0.5 µM) and rotenone/antimycin A (0.5 µM). The Glycolysis Stress test was used to measure ECAR. At 24 h post-seeding in miniplates, cells were incubated in assay medium lacking glucose for 1 hr. ECAR was measured in basal condition and following the step-wise addition of glucose, oligomycin, and 2-DG. For data normalization based on cell number, cells were fixed with 10% trichloroacetic acid, and cell density was determined using SRB assay.

Flow cytometry. Cells were stained with MitoTracker Green, JC-1 or 2-NBDG in PBS at 37ºC for 20-30 min followed by staining with DAPI [1]. Flow cytometry was done on FACS Canto (BD Biosciences). Data were analyzed using FlowJo (Tree Star).

Immunofluorescence microscopy. Cells were grown on glass coverslips and stained with MitoTracker Orange CMTMRos (ThermoFisher Scientific) for 40 min at 37C°C. After washing in PBS, cells were fixed with 4% paraformaldehyde and permeabilized with 0.1% Triton. Samples were mounted with Vectashield mounting medium containing DAPI. Images were obtained using Leica TCS SP5 confocal microscope.

Statistical analysis. Differences between groups were examined using two-tailed Student's t-test in normally distributed groups. *P* values of less than 0.05 were considered statistically significant. Statistical analysis was done using Graphpad Prism software.

References

1. Civenni, G.; Bosotti, R.; Timpanaro, A.; Vazquez, R.; Merulla, J.; Pandit, S.; Rossi, S.; Albino, D.; Allegrini, S.; Mitra, A., et al. Epigenetic Control of Mitochondrial Fission Enables Self-Renewal of Stem-like Tumor Cells in Human Prostate Cancer. *Cell Metab* **2019**, *30*, 303-318 e306, doi:10.1016/j.cmet.2019.05.004.
2. Anders, S.; Huber, W. Differential expression analysis for sequence count data. *Genome Biol* **2010**, *11*, R106, doi:10.1186/gb-2010-11-10-r106.
3. Brock, G.; Puihur, V.; Datta, S.; Datta, S. clValid: An R Package for Cluster Validation. *Journal of Statistical Software* **2008**, *25*, 1-22.

4. Colaprico, A.; Silva, T.C.; Olsen, C.; Garofano, L.; Cava, C.; Garolini, D.; Sabedot, T.S.; Malta, T.M.; Pagnotta, S.M.; Castiglioni, I., et al. TCGAbiolinks: an R/Bioconductor package for integrative analysis of TCGA data. *Nucleic Acids Res* **2016**, *44,* e71, doi:10.1093/nar/gkv1507.

5. Mounir, M.; Lucchetta, M.; Silva, T.C.; Olsen, C.; Bontempi, G.; Chen, X.; Noushmehr, H.; Colaprico, A.; Papaleo, E. New functionalities in the TCGAbiolinks package for the study and integration of cancer data from GDC and GTEx. *PLoS Comput Biol* **2019**, *15,* e1006701, doi:10.1371/journal.pcbi.1006701.

6. Silva, T.C.; Colaprico, A.; Olsen, C.; D'Angelo, F.; Bontempi, G.; Ceccarelli, M.; Noushmehr, H. TCGA Workflow: Analyze cancer genomics and epigenomics data using Bioconductor packages. *F1000Res* **2016**, *5*, 1542, doi:10.12688/f1000research.8923.2.

Supplementary Figure legends

Supplementary Figure S1. Response of human HCC cell lines to sorafenib. (A) Proliferation of human HCC cell lines incubated with sorafenib. Cell viability was measured after 72 h by the MTT assay. (B) Growth of HepG2, Huh7, SNU-387, and SNU-423 exposed to sorafenib in colony-forming assays.

Supplementary Figure S2. Growth properties of parental and sorafenib resistant cells. (A) Quantification of colony formation by parental (left panel) and sorafenib-resistant (right panel) Huh7 cells exposed to sorafenib shown in Figure 1B. (B) Proliferation of parental and sorafenib-resistant (Sorafenib-R1 and R2) Huh7 cells in adherent cell culture conditions determined by cell counting. (C) Tumor-sphere forming ability of parental and sorafenib-resistant Huh7 cells in 3D stem cell-selective culture conditions. (D) Growth of subcutaneous xenografts of parental and sorafenib-resistant Huh7 cells in nude mice (n=10 mice/group). Right panel, mean tumor weight at the end of the experiment.

Supplementary Figure S3. MRP expression in HCC patients. (A) Distribution according to stage and MRP-based clustering of patients in the total cohort (Top) and in the subset of patients treated with sorafenib (Bottom) in the TCGA dataset. (B) Differential clustering of HCC and normal liver samples in the TCGA cohort and cumulative MRP gene set expression. Total numbers of normal liver and HCC samples are 50 and 340. Bottom, p-values for all class comparisons. (C) Differential clustering of HCC samples in the ICGC patient cohort and cumulative MRP gene set expression. Total number of HCC samples included in the analysis is 240. Bottom, p-values for all class comparisons. (D) MRP-based clusters and overall survival in sorafenib-treated patients in the

TCGA cohort. (E-F) Cox regression multivariate survival analysis considering MRP gene expression, clustering and stage in the entire TCGA cohort (E) and the sorafenib-treated patients (F).

Supplementary Figure S4. Mitochondrial ribosomal proteins and clinical outcome in HCC. Association of the expression of MRP genes and overall survival in the subset of sorafenib-treated HCC patients (A) and in the total HCC patient population (B) in the TCGA cohort by Cox proportional hazard regression analysis. MRP genes aligned by increasing p-value.

Supplementary Figure S5. Glycolytic metabolism in parental and sorafenib resistant Huh7 cells. (A) Extracellular acidification rate (ECAR) assessed using Seahorse XFp analyzer in parental and sorafenib-resistant Huh7 cells. Top, real-time OCR measurements normalized to cell number. Bottom, basal and spare respiratory capacity based on OCR profiles. * $P \leq 0.05$. (B) Glucose uptake assessed by flow cytometry analysis of 2-NBDG-stained parental and sorafenib-resistant Huh7 cells.

Supplementary Figure S6. Mitochondrial metabolism in sorafenib-treated Huh7 cells. (A-B) Mitochondrial OCR in parental Huh7 cells treated with sorafenib (5-10 µM) for 6 h (A) and 4 days (B). *Top panels*, real-time OCR measurements normalized to cell number. *Bottom panels*, basal and spare respiratory capacity based on OCR profiles.

Supplementary Figure S7. JAK/STAT3 pathway in sorafenib-resistant cells. (A-B) Presence of IL6/JAK/STAT3 regulated genes (A) and STAT3 target genes (B) among up- and down- regulated genes in sorafenib-resistant cells.

Supplementary Figure S8. Impact of STAT3 depletion on sorafenib resistance. (A) Quantification of colony formation by sorafenib-resistant cells treated with sorafenib with and without induction of STAT3 knockdown by IPTG as shown in Figure 3D. (B-C) Mitochondrial membrane potential by JC1 staining (B) and mitochondrial mass by MitoTracker Green staining (C) in sorafenib-resistant Huh7 cells expressing inducible shRNA targeting STAT3. Cells were treated with IPTG (5 mM) for 7 days prior to flow cytometry analysis. (D) Mitochondrial OCR in sorafenib-resistant Huh7 cells with and without inducible STAT3 knockdown. Cells were treated for 7 days with IPTG (5 mM) prior to mitochondrial stress test and OCR measurement by Seahorse XFp analyzer.

Supplementary Figure S9. Effects of STAT3 inhibition on sorafenib resistance. (A) Quantification of colony formation by sorafenib-resistant cells treated with sorafenib and OPB-111077 as shown in Figure 5A. (B) Quantification of colony formation by sorafenib-resistant cells treated with sorafenib and metformin as shown in Figure 5F.

Supplementary Table legends

Supplementary Table S1. Gene ontology (GO) enrichment analysis of cellular components. Comparison of genes upregulated (top) and downregulated (bottom) between sorafenib-resistant and parental cells (adjusted p-value ≤ 0.05).

Supplementary Table S2. Putative Myc/Max targets among genes upregulated in sorafenib-resistant cells.

Supplementary Table S3. Putative SOX2 targets among genes downregulated in sorafenib-resistant cells.

Supplementary Table S4. Gene set enrichment analysis of drug perturbations in sorafenib-resistant cells. Comparative analysis of genes upregulated and downregulated in sorafenib-resistant cells versus parental cells (adjusted p-value ≤ 0.005).

Supplementary Dataset legends

Supplementary Data set S1. Upregulated genes in sorafenib-resistant Huh7 cells. Genes with FC ≥ 1.4 and p-value ≤ 0.05.

Supplementary Data set S2. Downregulated genes in sorafenib-resistant Huh7 cells. Genes with FC ≥ 1.4 and p-value ≤ 0.05.
