# Supplementary material for: Mitochondrial Plasticity Promotes Resistance to Sorafenib and Vulnerability to STAT3 Inhibition in Human Hepatocellular Carcinoma
Source: Cancers (Basel). 2021 Nov 30;13(23):6029. doi: 10.3390/cancers13236029 (PMC8657239; doi:10.3390/cancers13236029)
Supplement: Supplementary file 1 [file cancers-13-06029-s001.zip › Supplementary original image file.pdf]

Figure 3E

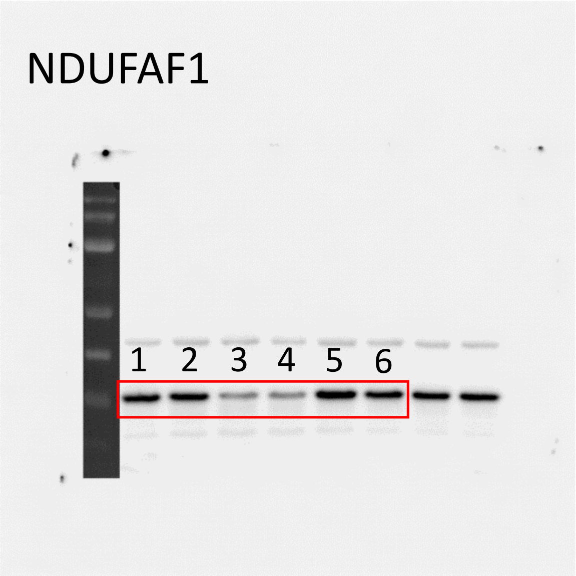

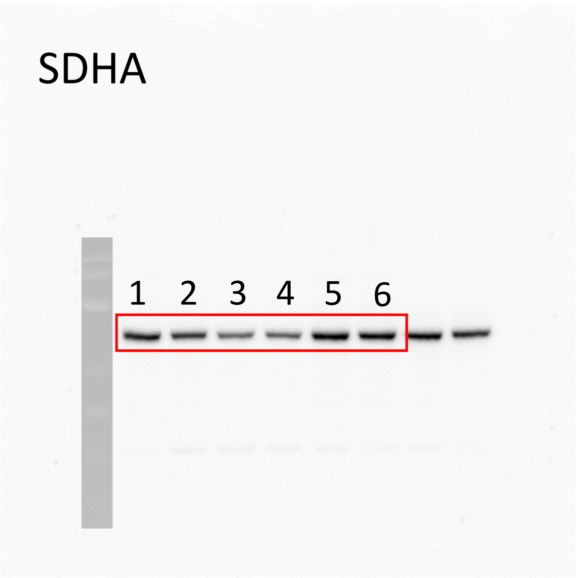

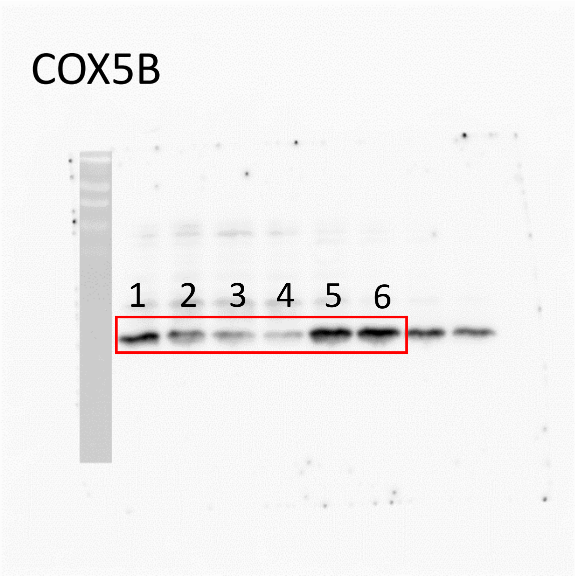

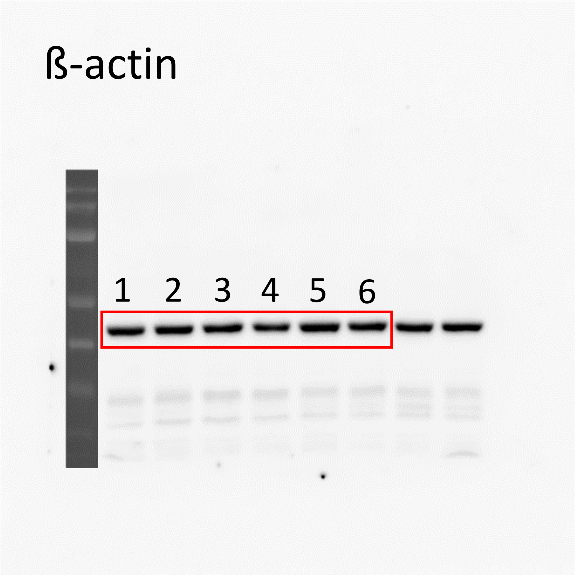

Figure 3F

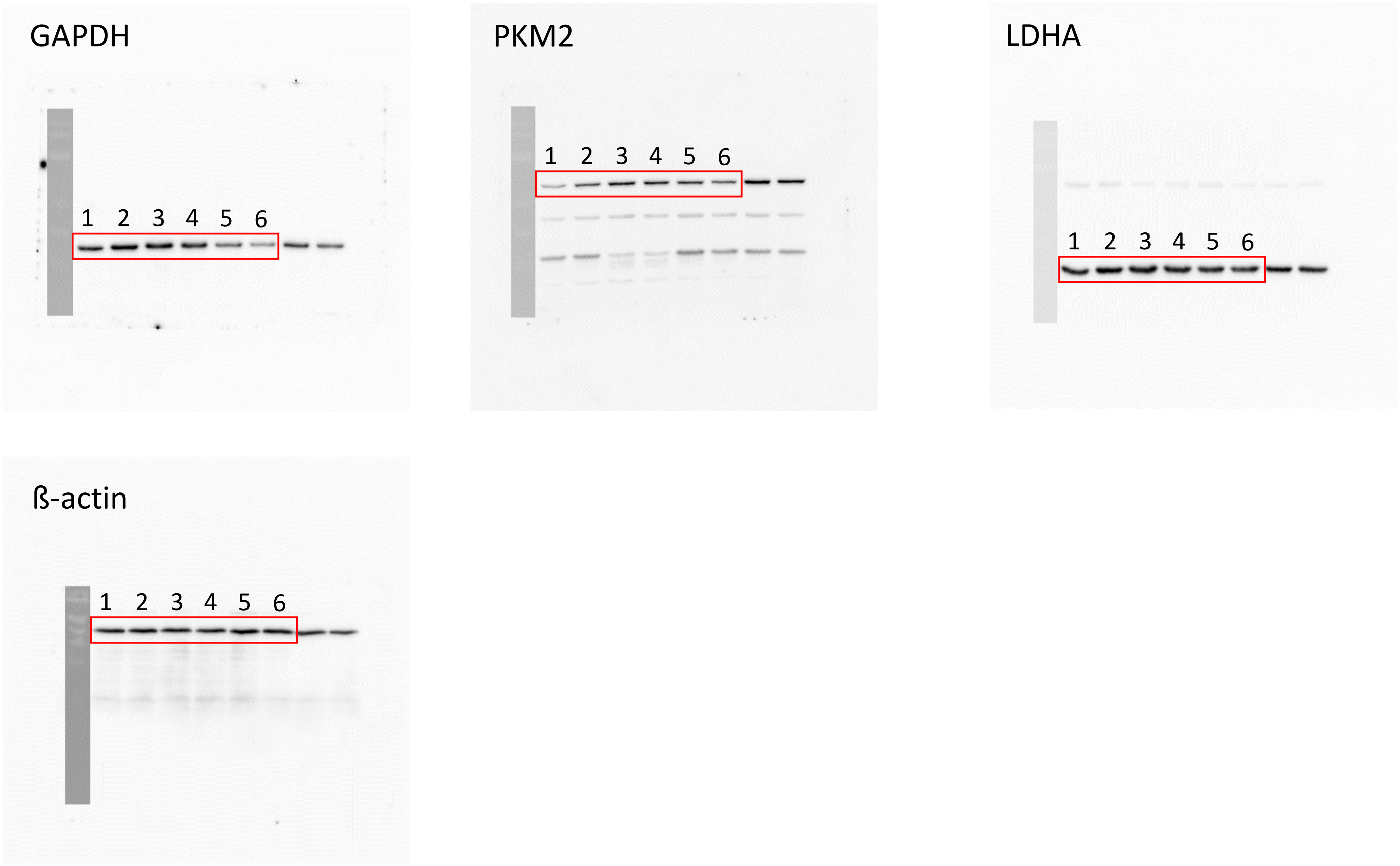

Figure 3G

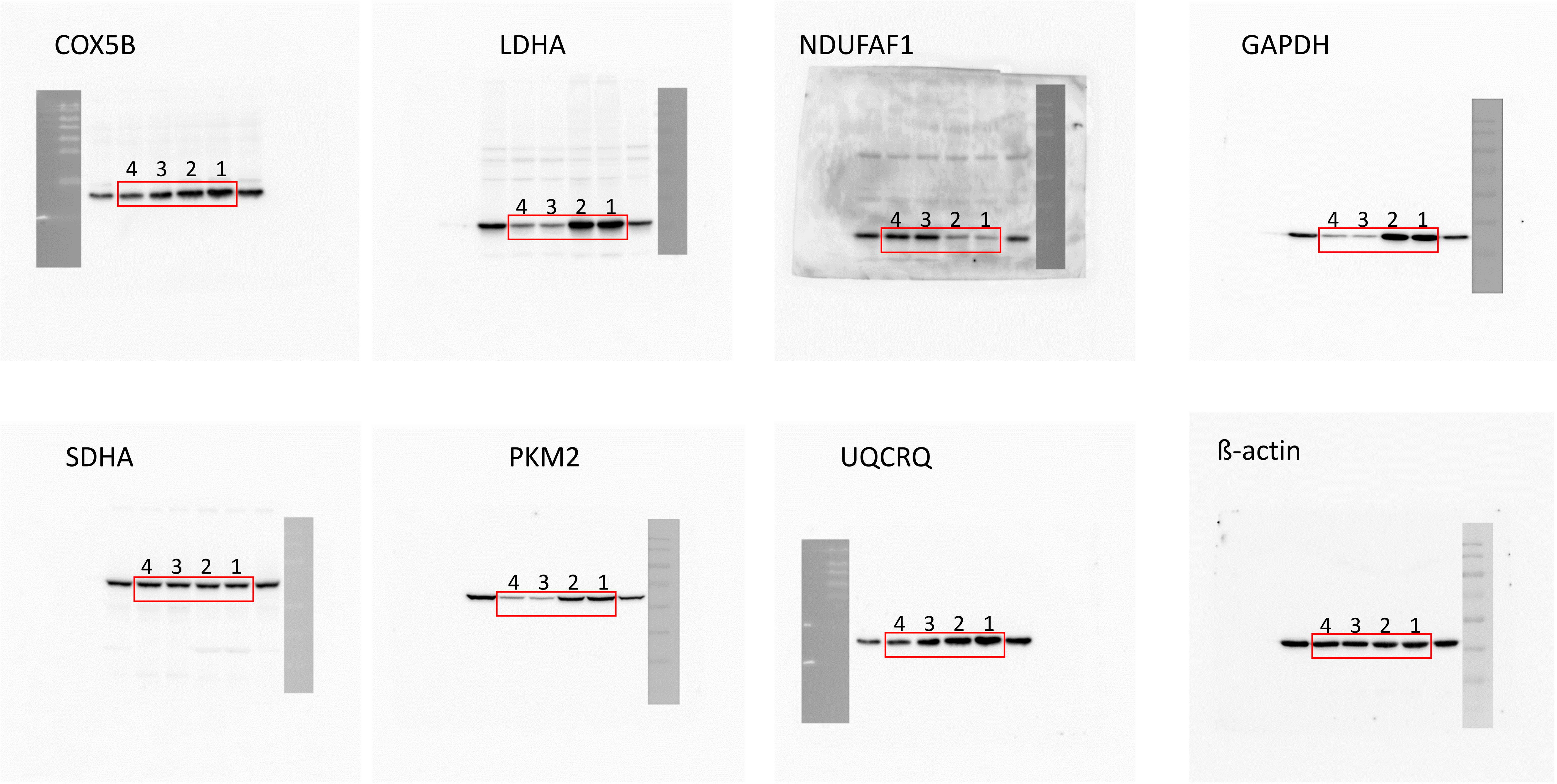
COX5B
4 3 2 1
LDHA
4 3 2 1
NDUFAF1
4 3 2 1
GAPDH
4 3 2 1
SDHA
4 3 2 1
PKM2
4 3 2 1
UQCRQ
4 3 2 1
ß-actin
4 3 2 1

Figure 4A

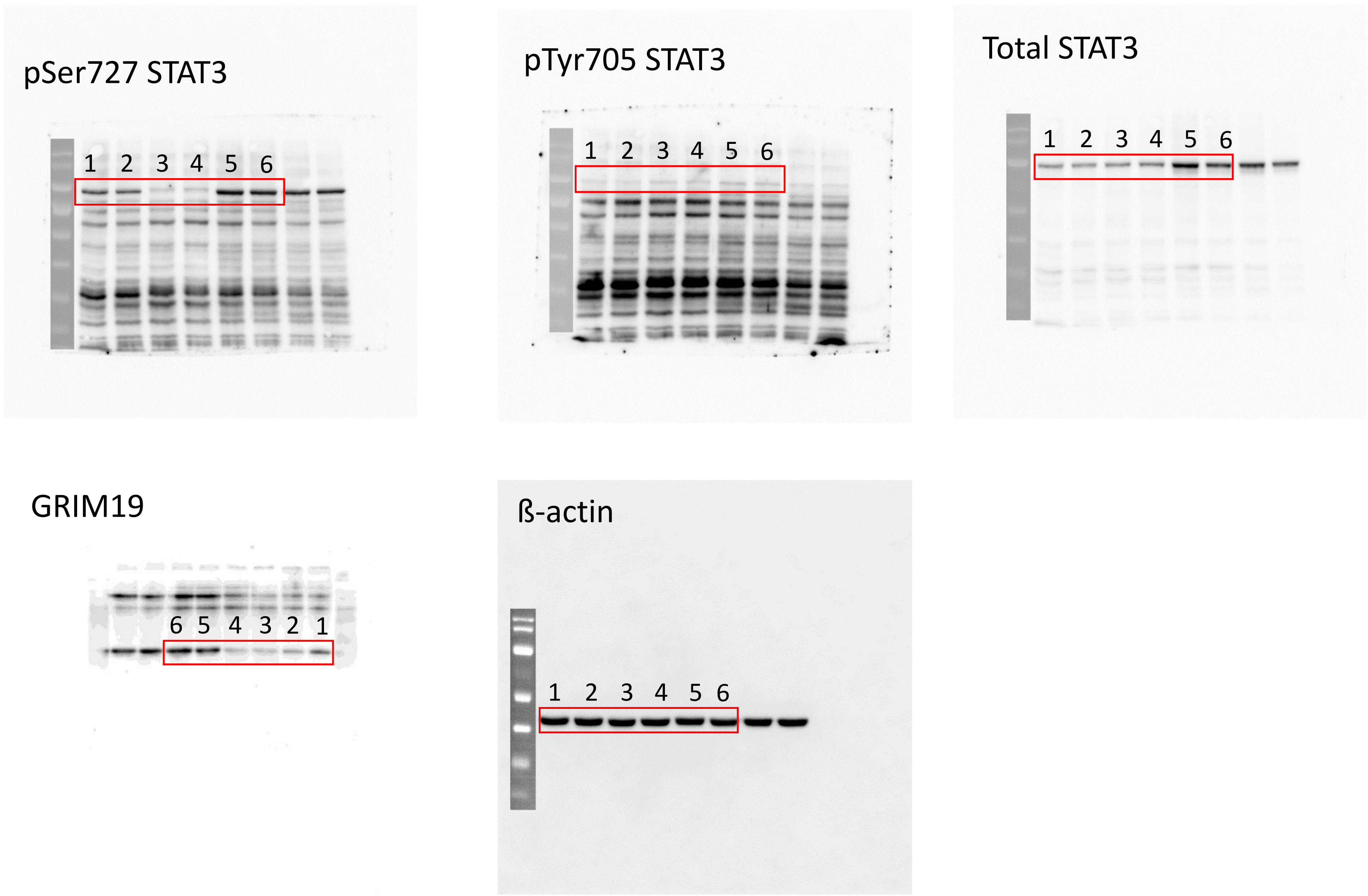

pSer727 STAT3
1 2 3 4 5 6
pTyr705 STAT3
1 2 3 4 5 6
Total STAT3
1 2 3 4 5 6
GRIM19
6 5 4 3 2 1
ß-actin
1 2 3 4 5 6

Figure 4B

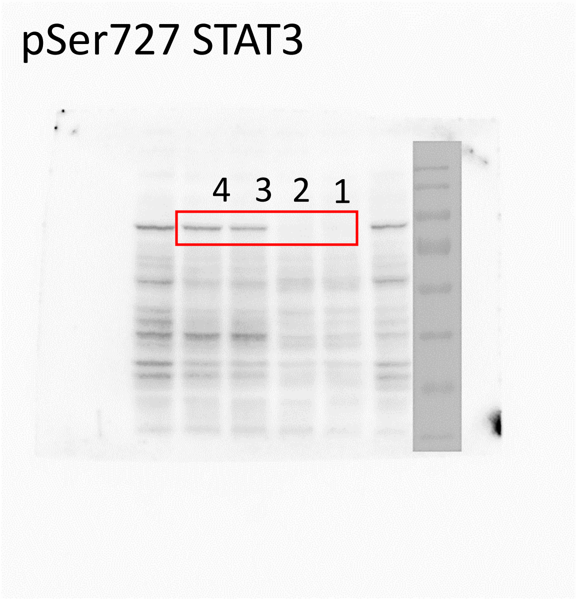
pSer727 STAT3
4 3 2 1

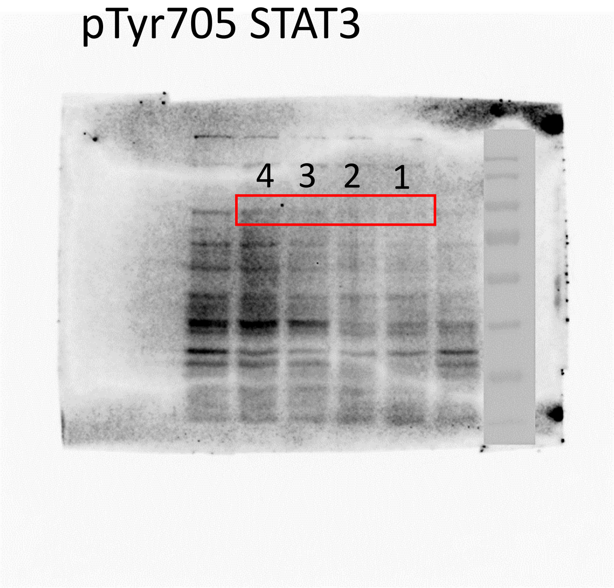
pTyr705 STAT3
4 3 2 1

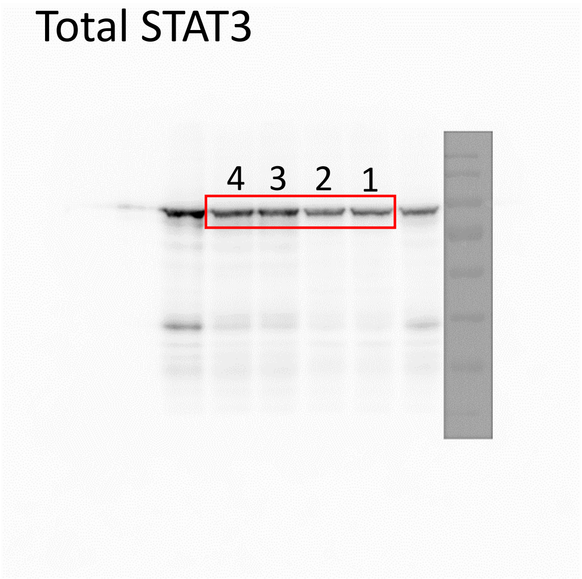
Total STAT3
4 3 2 1

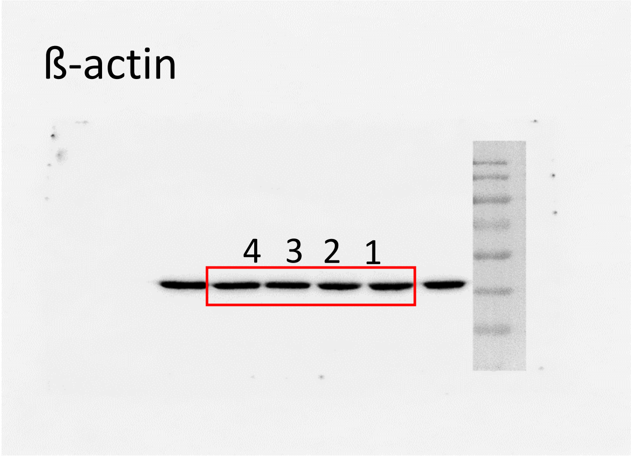
ß-actin
4 3 2 1

Figure 4C

Total STAT3

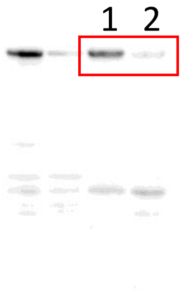

ß-actin

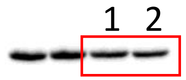

Figure 5B

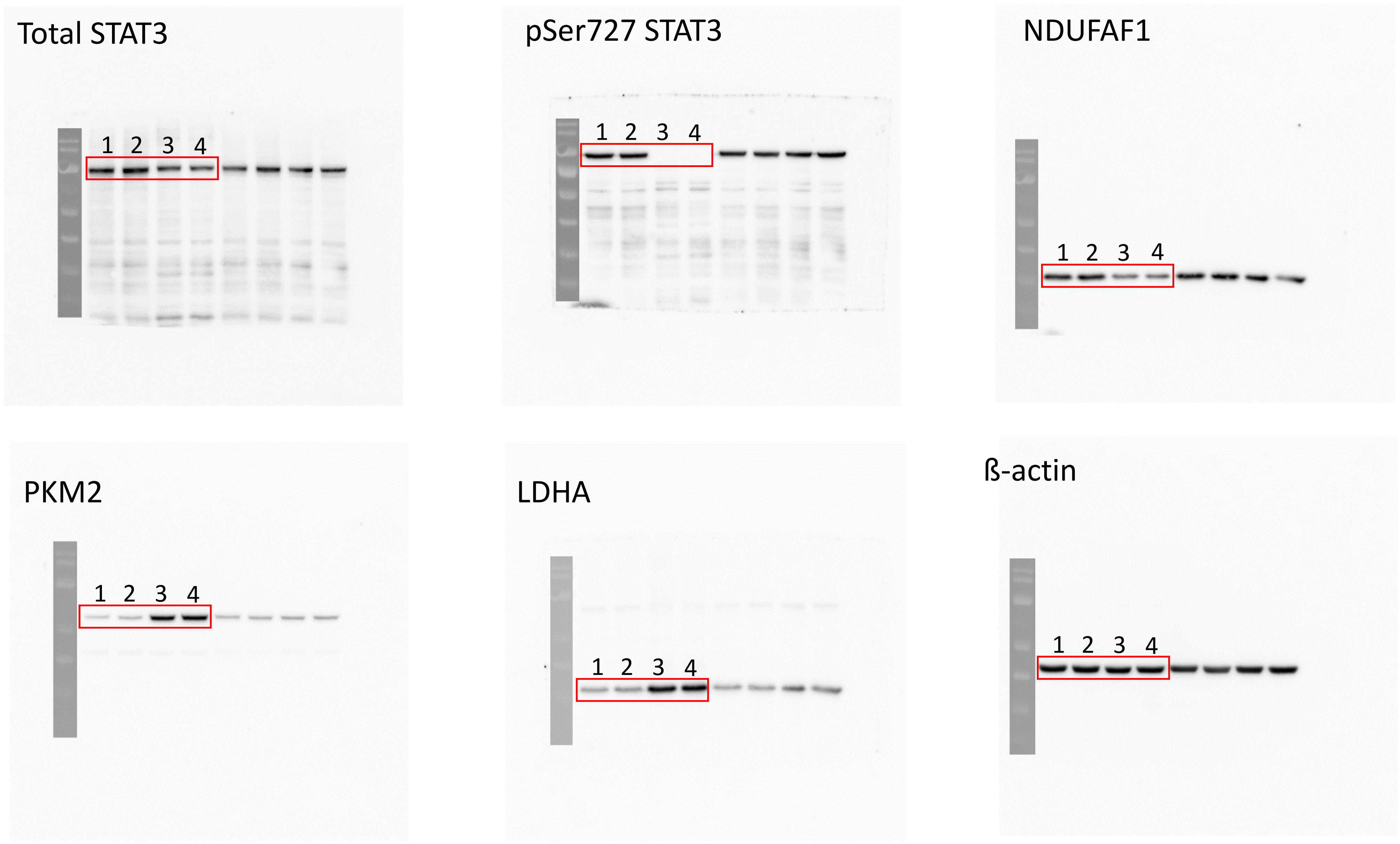
Total STAT3
1 2 3 4
pSer727 STAT3
1 2 3 4
NDUFAF1
1 2 3 4
PKM2
1 2 3 4
LDHA
1 2 3 4
ß-actin
1 2 3 4
