# Supplementary material for: Mitochondrial Plasticity Promotes Resistance to Sorafenib and Vulnerability to STAT3 Inhibition in Human Hepatocellular Carcinoma
Source: Cancers (Basel). 2021 Nov 30;13(23):6029. doi: 10.3390/cancers13236029 (PMC8657239; doi:10.3390/cancers13236029)
Supplement: Supplementary file 1 [file cancers-13-06029-s001.zip › Supplementary table S1-Cell components.pdf]

Supplementary table S1. Gene Ontology cellular component enrichment in sorafenib-resistant Huh7 cells

Index	Name	*p*-value	Adjusted *p*-value [3]	Z-score	Combined score
		**Upregulated Genes[1]**			
1	Nucleolus	0.000002684	0.0007355	-1.57	20.16
2	Preribosome	0.000152	0.020768	-1.37	12.06
3	Mitochondrion	0.0002708	0.02473	-1.82	14.96

Index	Name	*p*-value	Adjusted *p*-value	Z-score	Combined score
		**Downregulated genes[2]**			
1	Endoplasmic reticulum lumen	2.462e-7	0.00003213	-1.54	23.38
2	Platelet alpha granule	5.988e-7	0.00005209	-1.62	23.20
3	Platelet alpha granule lumen	0.000009623	0.0004186	-1.93	22.32
4	Vacuolar lumen	0.0001403	0.004577	-2.13	18.89
5	Focal adhesion	1.324e-7	0.00003213	-1.13	17.93
6	Azurophil granule	0.000001974	0.0001288	-1.35	17.80

[1]Genes upregulated in sorafenib-resistant Huh7 cellscompared to parental huh7 cells. [2]Genes downregulated in sorafenib-resistant cells compared to parental Huh7 cells. [3]Adjusted *p*-value by the Benjamini–Hochberg method.
