# Supplementary material for: Mitochondrial Plasticity Promotes Resistance to Sorafenib and Vulnerability to STAT3 Inhibition in Human Hepatocellular Carcinoma
Source: Cancers (Basel). 2021 Nov 30;13(23):6029. doi: 10.3390/cancers13236029 (PMC8657239; doi:10.3390/cancers13236029)
Supplement: Supplementary file 1 [file cancers-13-06029-s001.zip › Supplementary table S4-Drug perturbations.pdf]

Supplementary table S4. Enrichment analysis of drug perturbation gene sets in sorafenib-resistant Huh7 cells

Upregulated genes[1] - Drug perturbations from GEO: Downregulated gene sets

Index	Name	*p*-value	Adjusted *p*-value[3]	Z-score	Combined score
1	**vemurafenib DB08881 human GSE37441 sample 2563**	7.740e-16	6.989e-13	-1.63	56.65
2	**vemurafenib DB08881 human GSE37441 sample 2564**	3.825e-13	1.727e-10	-1.63	46.68
3	4-Hydroxynonenal 5283344 human GSE2397 sample 3078	4.872e-11	1.466e-8	-1.67	39.55
4	**vemurafenib DB08881 human GSE37441 sample 2560**	1.052e-10	1.900e-8	-1.63	37.40
5	Curcumin 969516 human GSE16160 sample 3425	7.906e-11	1.785e-8	-1.59	36.97
6	doxorubicin, EC50, 1 d 31703 human GSE6930 sample 3260	3.519e-8	0.000005296	-1.70	29.13
7	RPI-1 1749978 human GSE49414 sample 3173	1.153e-7	0.00001487	-1.61	25.74
8	Promyelocytic leukemia DB00755 human GSE5007 sample 2461	4.567e-7	0.00003758	-1.73	25.21
9	cytarabine, 2xEC50, 1 d 6253 human GSE6930 sample 3422	5.179e-7	0.00003758	-1.71	24.72

Upregulated genes[1] - Drug perturbations from GEO: Upregulated gene sets

Index	Name	p-value	Adjusted p-value[3]	Z-score	Combined score
1	**cetuximab DB00002 human GSE61495 sample 3135**	1.282e-16	1.160e-13	-1.73	63.14
2	**cetuximab DB00002 human GSE61495 sample 3136**	6.597e-11	2.985e-8	-1.59	37.38
3	doxycycline DB00254 human GSE2624 sample 3077	2.940e-7	0.00008869	-1.63	24.47
4	motexafin gadolinium (12 h) DB05428 human GSE2189 sample 3127	0.000001605	0.0003632	-1.68	22.42
5	estradiol DB00783 human GSE11352 sample 2729	0.000008530	0.001544	-1.85	21.56
6	progesterone 5994 mouse GSE6440 sample 3686	0.00004735	0.006122	-1.66	16.55
7	doxycycline DB00254 human GSE2624 sample 3074	0.00004708	0.006122	-1.57	15.69
8	chromium 23976 human GSE6907 sample 3534	0.0001109	0.01255	-1.71	15.54
9	estradiol 5757 human GSE16683 sample 3394	0.0003842	0.03359	-1.80	14.19
10	estradiol 5757 human GSE53394 sample 2652	0.0003319	0.03337	-1.71	13.73

Downregulated genes[2] - Drug perturbations from GEO: Downregulated gene sets

Index	Name	*p*-value	Adjusted *p*-value[3]	Z-score	Combined score
1	trovafloxacin DB00685 human GSE9166 sample 3036	2.426e-20	2.188e-17	-1.63	73.49
2	HYPOCHLOROUS ACID 24341 human GSE11630 sample 3202	3.972e-19	9.543e-17	-1.69	71.75
3	lung cancer DB00928 human GSE29077 sample 2535	1.504e-19	6.782e-17	-1.64	71.24
4	lung cancer DB00928 human GSE29077 sample 2536	4.232e-19	9.543e-17	-1.67	70.70
5	**cetuximab DB00002 human GSE61495 sample 3136**	4.230e-18	7.630e-16	-1.58	63.29
6	Harman 5281404 human GSE5080 sample 3482	2.157e-17	3.242e-15	-1.61	61.72
7	VX 39793 human GSE33606 sample 3376	1.151e-15	1.483e-13	-1.63	56.06
8	hydrocortisone DB00741 human GSE7890 sample 2751	2.463e-15	2.777e-13	-1.65	55.40
9	estradiol 5757 human GSE26834 sample 3241	5.053e-15	4.558e-13	-1.64	54.03
10	lung cancer DB00928 human GSE29077 sample 2534	4.533e-15	4.543e-13	-1.62	53.66

Downregulated genes[2] - Drug perturbations from GEO: Upregulated gene sets

Index	Name	*p*-value	Adjusted *p*-value[3]	Z-score	Combined score
1	BPDE 41322 human GSE19510 sample 3379	3.240e-24	2.933e-21	-1.72	93.20
2	**vemurafenib DB08881 human GSE37441 sample 2561**	3.506e-20	1.586e-17	-1.68	75.09
3	metoprolol DB00264 human GSE3356 sample 2786	1.963e-16	4.441e-14	-1.94	70.17
4	5-aza-2'-deoxycytidine DB01262 human GSE5230 sample 2497	1.280e-16	3.861e-14	-1.84	67.18
5	BPDE 41322 human GSE19510 sample 3378	1.200e-14	1.358e-12	-1.82	58.23
6	gamma-Tocotrienol 5282349 human GSE21946 sample 3242	6.369e-15	1.045e-12	-1.77	57.81
7	cytarabine, 2xEC50, 3 d 6253 human GSE6930 sample 3423	8.083e-15	1.045e-12	-1.66	53.82
8	letrozole 3902 human GSE5462 sample 3097	1.801e-14	1.811e-12	-1.65	52.31
9	resveratrol DB02709 human GSE25412 sample 3500	7.843e-15	1.045e-12	-1.60	52.12
10	tibolone 444008 human GSE12446 sample 3204	1.184e-12	1.010e-10	-1.70	46.76

[1]Genes upregulated in sorafenib-resistant Huh7 cells compared to parental huh7 cells. [2]Genes downregulated in sorafenib-resistant cells compared to parental Huh7 cells. [3]Adjusted *p*-value by the Benjamini–Hochberg method.
